# Acyclic cucurbit[*n*]uril bearing alkyl sulfate ionic groups

**DOI:** 10.3762/bjoc.21.55

**Published:** 2025-04-03

**Authors:** Christian Akakpo, Peter Y Zavalij, Lyle Isaacs

**Affiliations:** 1 Department of Chemistry and Biochemistry, University of Maryland, College Park, Maryland 20742, United States

**Keywords:** cucurbituril, host–guest chemistry, isothermal titration calorimetry, molecular container, X-ray crystallography

## Abstract

We report the synthesis and characterization of a new acyclic cucurbit[*n*]uril (CB[*n*]) host **C1** that features four alkyl sulfate ionic groups. The X-ray crystal structure of the **C1·Me_6_CHDA** complex is reported. Host **C1** is significantly less soluble in water (4 mM) compared to the analogous acyclic CB[*n*] host **M1** which features sulfonate ionic groups (346 mM). Host **C1** does not undergo significant self-association according to the results of ^1^H NMR dilution experiments. The molecular recognition behavior of the hosts **C1** and **M1** toward a panel of seven ammonium ions was explored by ^1^H NMR spectroscopy and isothermal titration calorimetry (ITC). We find that **C1** generally binds slightly more tightly than **M1** toward a specific guest. **C1** binds more tightly to quaternary ammonium guests compared to the corresponding primary ammonium ions.

## Introduction

Molecular recognition interactions are key elements of life processes including self- versus non-self-recognition, biosynthesis, molecular and ion transport, and replication. Beginning with the pioneering works of Pedersen, Lehn, and Cram, supramolecular chemists have studied the fundamental aspects of non-covalent interactions in organic solvents and water [1–4]. Building on this fundamental knowledge, supramolecular chemists created a variety of functional systems including supramolecular polymers, sensing ensembles, molecular machines, supramolecular separation phases, and drug delivery systems [5–9]. A primary subfield of supramolecular chemistry involves the synthesis of macrocyclic hosts and studies of their molecular recognition properties. The most widely studied macrocyclic host systems include those created entirely by covalent bonds (crown ethers, cyclodextrins, calixarenes, cyclophanes, pillararenes, cucurbit[*n*]urils (CB[*n*])), and those prepared by metal ligands and H-bonding self-assembly processes [1–2,10–20]. Macrocycles have played key roles in important real-world products including the household deodorizer Febreeze^TM^, glucose monitors, and as solubilizing excipients [21–26]. Within these families of macrocyclic hosts, CB[*n*] molecular containers have proven particularly versatile because they form high affinity CB[*n*]–guest complexes in aqueous solution that are responsive to various stimuli (e.g., photochemical, electrochemical, chemical) [27–30]. For this reason, macrocyclic CB[*n*] have been used as key elements of separations processes [31–32], sensing systems [33–34], in pharmaceutical applications [35–38], in bioimaging systems [39–40], and even in household deodorizing products [41].

**Figure 1:**
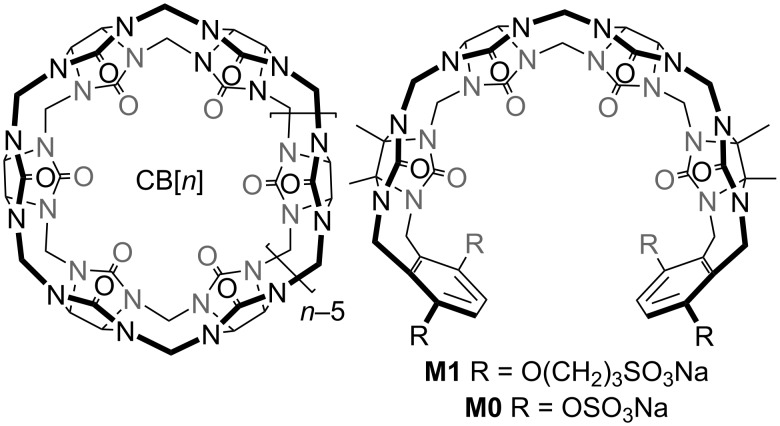
Chemical structures of CB[n] and selected acyclic CB[*n*]-type molecular containers **M1** and **M0**.

An important subclass of CB[*n*] hosts are acyclic CB[*n*]-type receptors which have been extensively studied by our lab and others over the past decade [42–52]. Figure 1 shows the chemical structure of the prototypical acyclic CB[*n*]-type known as **M1** [53–54]. **M1** features a central glycoluril tetramer, two aromatic *o*-xylylene walls, and four sulfonates as solubilizing ionic groups. In accord with these structural features, **M1** binds a variety of hydrophobic and cationic guest molecules by the hydrophobic effect, π–π interactions, and electrostatic (ion–dipole and ion–ion) interactions. Although acyclic CB[*n*] are not macrocycles, they are preorganized into a C-shaped geometry by virtue of their polycyclic chemical structure and display binding affinities approaching those of macrocyclic CB[*n*]. **M1** and analogues display outstanding biocompatibility and have been used for a number of in vivo biomedical applications including as a solubilizing excipient for anticancer agents and as an in vivo sequestrant to reverse the biological activity of neuromuscular blocking agents, anesthetics, and drugs of abuse (e.g., methamphetamine and fentanyl) [54–60].

As a result of their modular synthesis, acyclic CB[*n*] can be easily modified synthetically [42–47,61]. Acyclic CB[*n*]-type receptors featuring different length glycoluril oligomers (monomer–pentamer) and different aromatic walls (e.g., naphthalene, anthracene, triptycene) have been studied [42,62–67]. Previously, we have studied the influence of the length of the O(CH_2_)*_n_*SO_3_Na sidearm (*n* = 0, 2, 3, 4) and found that the **M0** host – where the hydrophobic linker (CH_2_)*_n_* was completely removed – displayed higher binding affinity than **M1** which we attributed to the location of the ionic group closer to the ureidyl C=O portals [68–69]. However, a close examination of the structures of **M0** and **M1** show that the ionic group for **M1** is a sulfonate and for **M0** is a sulfate. Accordingly, **M1** and **M0** differ in two ways: a) different (CH_2_)*_n_* linker length and b) different ionic group (sulfonate versus sulfate). In this paper, we present the synthesis and molecular recognition properties of a new acyclic CB[*n*]-type receptor **C1** which allows us to disentangle these two effects.

## Results and Discussion

This results and discussion section is organized as follows: First, we present the design, synthesis, and spectroscopic characterization of **C1** along with determination of its inherent aqueous solubility and self-association properties. Next, we present the X-ray crystal structure of **C1** as its **C1**·**Me_6_CHDA** complex. Subsequently, we describe a qualitative investigation of **C1**·guest and **M1**·guest complexation by ^1^H NMR spectroscopy and quantitative investigation by isothermal titration calorimetry (ITC). Finally, we discuss the trends in binding affinity observed for **C1**·guest and **M1**·guest complexation.

### Design, synthesis and characterization of **C1**

**Scheme 1:**
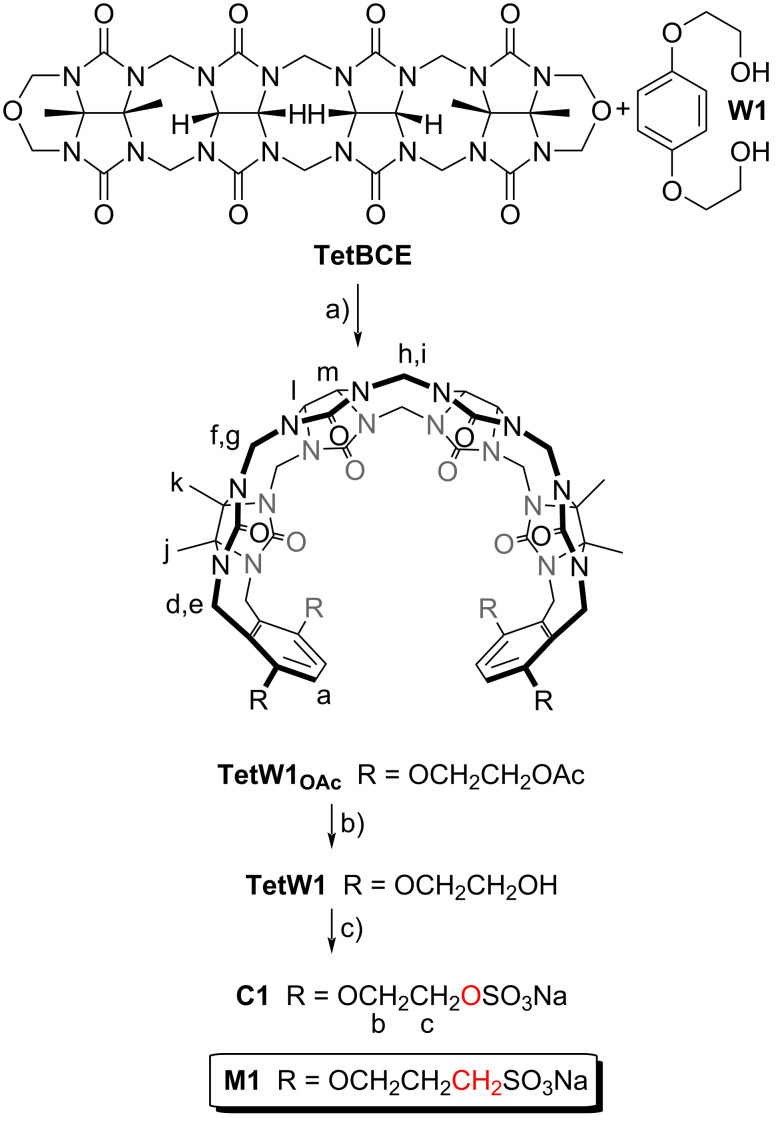
Synthesis of **C1**. Conditions: a) TFA/Ac_2_O, 70 °C, 3.5 h, 71%; b) LiOH, 50 °C, 69%; c) dry pyridine, pyridine sulfur trioxide complex (20 equiv), 90 °C, 18 h, 68%.

**Figure 2:**
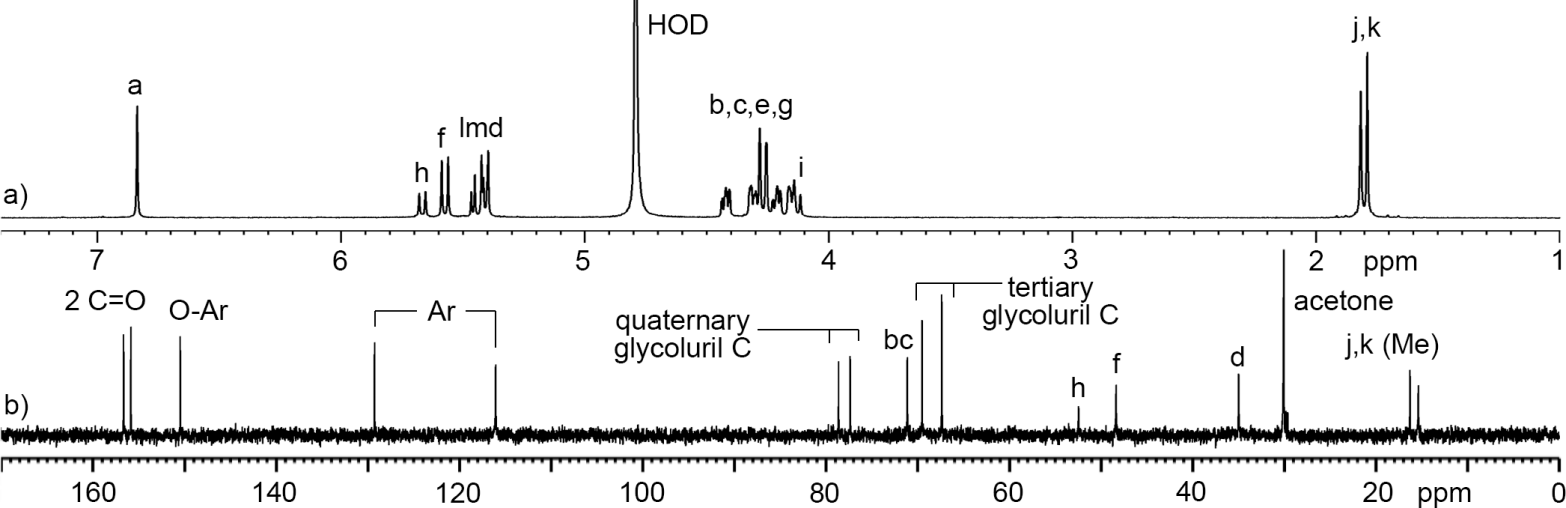
a) ^1^H NMR spectrum (600, D_2_O, rt) and b) ^13^C NMR spectrum recorded (150 MHz, D_2_O, rt) for **C1**.

In order to disentangle the effects of the ionic group (sulfonate versus sulfate) while maintaining the distance of the ionic group from the ureidyl C=O portal we designed acyclic CB[*n*]-type receptor **C1** (Scheme 1). The only structural difference between **M1** and **C1** is the swapping of one CH_2_ group for one O atom in each alkyl chain which effectively changes the sulfonate group to a sulfate group. The synthetic route to **C1** starts with the double electrophilic aromatic substitution reaction of methylene-bridged glycoluril tetramer (**TetBCE**) with **W1** in TFA/Ac_2_O 1:1 which adds the sidewalls and transforms the OH groups into OAc groups to give **TetW1_OAc_** in 71% yield as described previously [70]. Saponification of **TetW1_OAc_** with LiOH at 50 °C followed by acidification with 0.1 M HCl gives **TetW1** in 69% yield [70]. Finally, the sulfation of **TetW1** occurs upon treatment with py·SO_3_ (20 equiv) in dry pyridine to yield **C1** as a white solid in 68% yield. In accord with the depicted *C*_2_*_v_*-symmetric geometry (Scheme 1), the ^1^H NMR spectrum of **C1** displays one aromatic resonance (H_a_), two methyl resonances (CH_3_)_j_ and (CH_3_)_k_, two equatorial methine doublets (H_l_ and H_m_), along with three doublets for the diastereotopic methylene bridges around 5.5 ppm (H_d_, H_f_, H_h_) in the expected 2:2:1 ratio (Figure 2a). The 4.0–4.5 ppm region is crowded which precludes precise assignments of the expected resonances for H_e_, H_g_, H_i_, H_b_, and H_c_. Similarly, the ^13^C NMR spectrum recorded for **C1** (Figure 2b) shows 15 of the 16 resonances expected based on time averaged *C*_2_*_v_*-symmetry in solution. For example, we observe two resonances for the C=O groups, three resonances for the aromatic C-atoms, two methyl resonances, three resonances for the bridging CH_2_ groups, and five of the six resonances for the sidearm (b and c) and equatorial glycoluril C-atoms. The negative-ion electrospray ionization mass spectrum shows an ion at *m*/*z* = 751.13 which corresponds to [**C1** − 2Na]^2−^.

### Inherent aqueous solubility of **C1**

After having firmly established the structure of **C1** we decided to determine its inherent aqueous solubility. For this purpose, we added an excess of solid **C1** to D_2_O and stirred the solution at room temperature overnight. Afterwards, the mixture was centrifuged (4400 rpm, 10 min) to pellet excess insoluble **C1**. An aliquot of the supernatant and a solution of dimethyl malonic acid as a non-binding internal standard of known concentration were transferred to an NMR tube followed by collection of a ^1^H NMR spectrum using a delay time between pulses of 20 seconds to ensure accurate integration. The inherent aqueous solubility of **C1** was determined to be 3.97 mM by comparison of the integrals for H_a_ of **C1** with that of the CH_3_-resonance for dimethyl malonic acid (Figure S5 in Supporting Information File 1).

### Qualitative study of **C1**·guest recognition properties by ^1^H NMR spectroscopy

**Figure 3:**
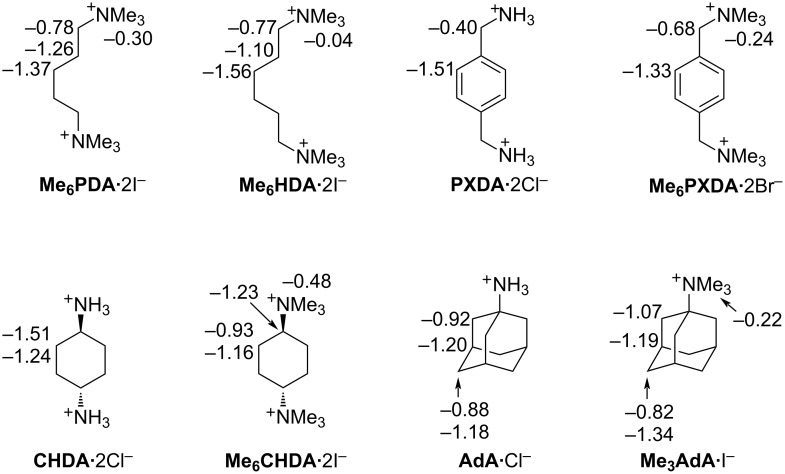
Chemical structures of guests used in this study along with the complexation induced changes in chemical shift (Δδ) upon formation of the **C1**·guest complexes. Negative Δδ values represent upfield shifts upon complexation.

**Figure 4:**
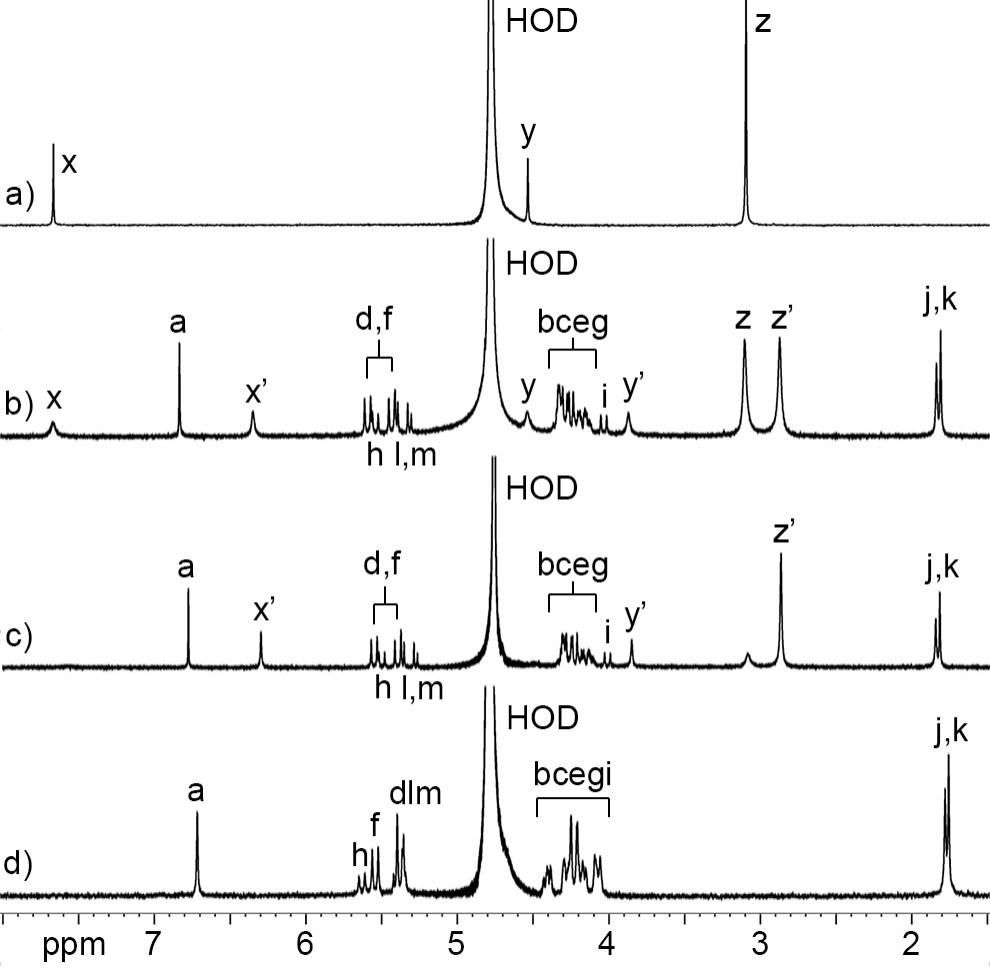
^1^H NMR spectra recorded (400 MHz, D_2_O, rt) for: a) **Me_6_PXDA** (0.5 mM), b) a mixture of **C1** (0.5 mM) and **Me_6_PXDA** (1.0 mM), c) a mixture of **C1** (0.5 mM) and **Me_6_PXDA** (0.5 mM), and d) **C1** (0.5 mM).

Next, we decided to perform a qualitative investigation of the host–guest properties of **C1** by ^1^H NMR spectroscopy. Figure 3 shows the chemical structures of a panel of guests that were studied and the complexation-induced changes in chemical shift (Δδ) for **C1**·guest. As the central hydrophobic binding domain of the guests we selected alkylene, *p*-xylylene, cyclohexane, and adamantane moieties that are known to bind well to (acyclic) CB[*n*] receptors [71–73]. The cross-sectional area of this hydrophobic moiety increases as follows: **PDA** ≈ **HDA** < **PXDA** < **CHDA** < **AdA**. Given that (acyclic) CB[*n*] often bind to ammonium ion guests (e.g., NH_3_^+^ form) weaker than they do to the corresponding methonium ion guests (e.g., NMe_3_^+^ form) we elected to study both forms to elucidate related preferences for the sulfated **C1** host relative to the sulfonated **M1** host [69,71,74]. Figure 4 shows a ^1^H NMR stack plot created for uncomplexed **C1** (Figure 4d), uncomplexed **Me_6_PXDA** (Figure 4d), and 1:1 and 1:2 mixtures of **C1** and **Me_6_PXDA**. Several spectroscopic features are noteworthy. First, the Ar–H resonance for **Me_6_PXDA** undergoes a large upfield shift (Δδ = −1.33) upon formation of **C1**·**Me_6_PXDA** (Figure 4c) whereas the CH_2_ (Δδ = −0.68) and NMe_3_ (Δδ = −0.24) groups undergo smaller upfield shifts. This observation strongly suggests that the Ar–H protons are located nearer the center of the magnetically shielding cavity of **C1** which is defined by the aromatic sidewalls and the ureidyl π-systems. The small changes in chemical shift for the methonium group suggests it is located near the ureidyl C=O portals and not inside the magnetically shielding cavity. Related complexation-induced changes in chemical shift are observed for the other **C1**·guest complexes (Figure 3 and Supporting Information File 1) which confirms that the hydrophobic central region of the guest binds inside the hydrophobic cavity of **C1** whereas the hydrophilic ammonium and methonium groups reside at the electrostatically negative ureidyl C=O portals. Second, at a 1:2 **C1**/**Me_6_PXDA** ratio (Figure 4b), we observe separate resonances for free **Me_6_PXDA** and complexed **C1**·**Me_6_PXDA** which means that the rate of guest exchange is slow on the chemical shift timescale. Slow kinetics of guest exchange is commonly observed for tight host·guest complexes. In contrast, the kinetics of guest exchange are in the intermediate exchange regime on the chemical shift timescale for the complexes of **C1** with **CHDA**, **Me_6_CHDA**, **AdA**, **Me_3_AdA** (Supporting Information File 1, Figures S10–S13) which is typical of weaker complexes. Third, we observe changes in the chemical shift for the H_a_ resonance of **C1** upon formation of the **C1**·guest complexes. In uncomplexed **C1** the tips of the aromatic rings are pointing toward each other which places H_a_ in the magnetically shielding region of the opposing sidewall. Upon formation of the **C1**·guest complexes, the tips of the aromatic sidewall change their orientation to accommodate the hydrophobic region of the guest which changes the orientation of H_a_ with respect to the magnetically shielding region [54,63–64].

### X-ray crystal structure of **C1**

**Figure 5:**
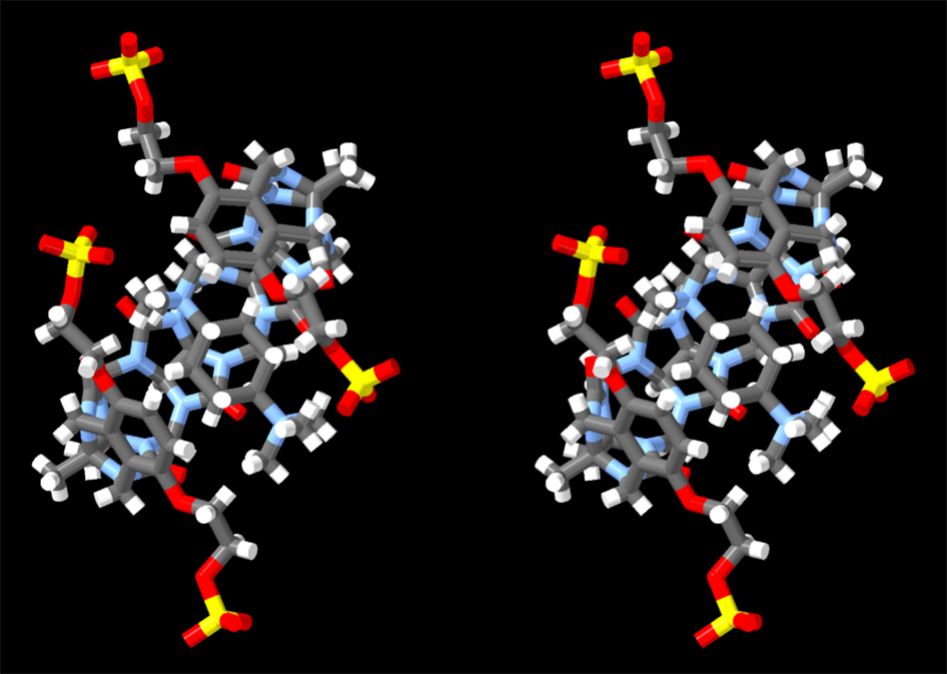
Cross-eyed stereoview of the **C1**·**Me_6_CHDA** complex in the crystal. Color code: C, gray; H, white; N, blue; O, red; S, yellow.

We were fortunate to obtain single crystals of the **C1**·**Me_6_CHDA** complex and solved the crystal structure by X-ray diffraction (CCDC 2411723). Figure 5 shows a cross-eyed stereoview of one **C1**·**Me_6_CHDA** complex in the crystal. Several features of this structure are noteworthy. First, the crystal structure confirms the molecular structure of **C1** and its overall C-shaped geometry. Second, within the **C1**·**Me_6_CHDA** complex, the aromatic sidewalls are splayed away from the equator of **C1** resulting in a helical geometry [63,65]. Both senses of helical chirality are present in the crystal; values in parenthesis given below refer to the complex with opposite helical chirality. The guest **Me_6_CHDA** possesses a mirror plane and is therefore achiral. In solution, host **C1** is flexible and the two senses of helicity – and other conformations – undergo rapid equilibrium rendering the **C1** and the **C1**·**Me_6_CHDA** complex achiral. The centroids of the aromatic sidewall are 0.9698 Å (1.1193 Å) above and 1.3090 Å (1.4832 Å) below the mean plane of the glycoluril methine and glycoluril quaternary C-atoms. Third, the **Me_6_CHDA** guest is not symmetrically oriented with respect to the ureidyl carbonyl portals of **C1**. Specifically, one of the methonium N-atoms is located inside the cavity of **C1** at 1.4476 Å (0.6162 Å) below the mean plane of the ureidyl carbonyl O-atoms whereas the other methonium N-atom is located 1.7980 Å (0.9686 Å) outside the cavity.

**Figure 6:**
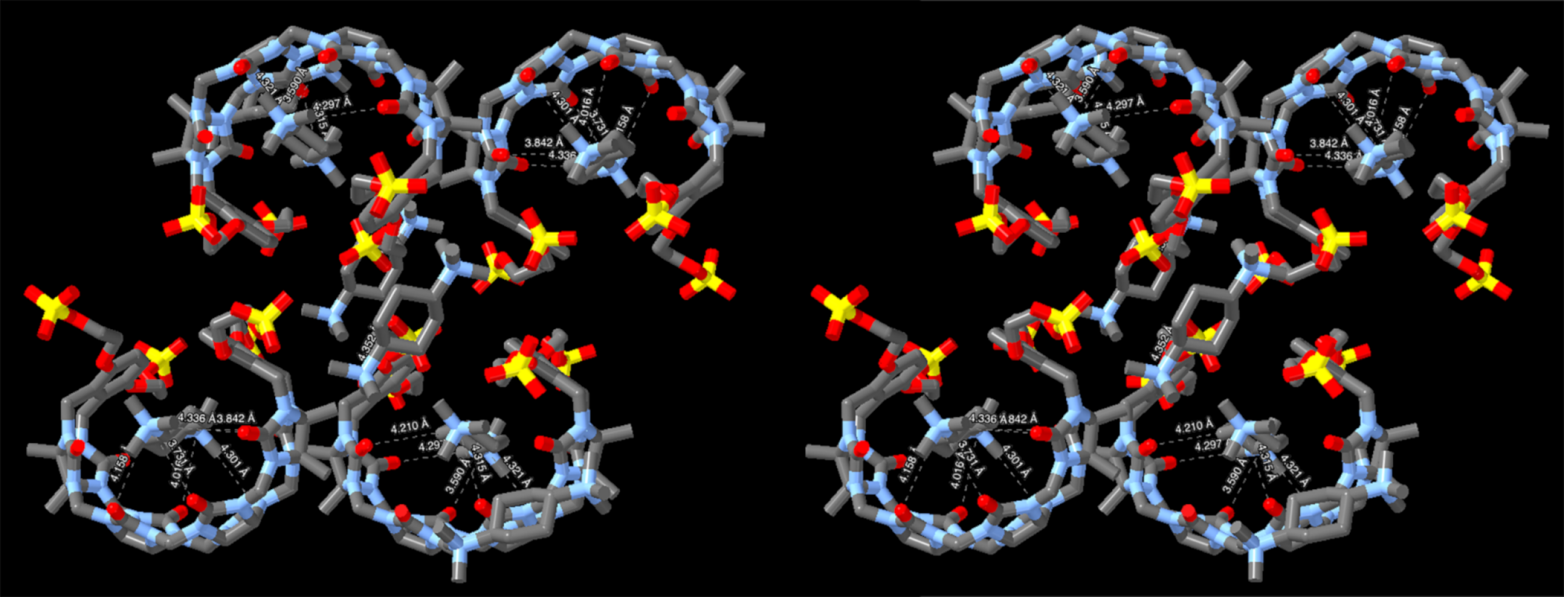
Cross-eyed stereoview of the crystal packing observed in the molecular cell of **C1**·**Me_6_CHDA**. H-atoms are omitted for clarity. N···O distances less than 4.40 Å are indicated with dashed lines. Color code: C, gray; N, blue; O, red; S, yellow.

Figure 6 shows the packing of four molecules of the **C1**·**Me_6_CHDA** complex in a single unit cell along with four molecules of **Me_6_CHDA** located outside the cavity of **C1** to ensure overall charge neutrality. It is well known that CB[*n*]·guest complexation is driven by ion–dipole interactions at the ureidyl C=O portals [75]. Previously, we found that the Me_3_N^+^···O=C distances in the ultratight CB[7]·diamantane(NMe_3_)_2_ complex averaged 4.38(7) Å [74]. For comparison, a histogram of Me_3_N^+^···O=C distances drawn from 89 CCDC structures that contain an acetylcholine-type unit (Me_3_NCH_2_CH_2_O(C=O)R) range from 3.5 Å to 5 Å with a maximum probability of 4.4 Å [74]. Figure 6 shows Me_3_N^+^···O=C contacts that are less than 4.40 Å. The large number of contacts that are significantly shorter than 4.40 Å establishes that Me_3_N^+^···O=C cation–dipole interactions play an important role driving the inclusion of **Me_6_CHDA** inside of **C1** to form the **C1**·**Me_6_CHDA** complex. Of course, the inclusion of the hydrophobic cyclohexyl moiety inside the cavity of **C1** provides a hydrophobic driving force for complexation in water. Given that **C1** is a tetraanion and that **Me_6_CHDA** is a dication, an additional molecule of **Me_6_CHDA** is present per molecule of **C1** to ensure overall charge neutrality in the crystal. Among the four molecules of **Me_6_CHDA** outside the cavity of **C1** in the molecular cell (Figure 6, only two external **Me_6_CHDA** are shown for clarity), only one Me_3_N^+^···O=C contact (4.548 Å) with a distance < 5.5 Å is observed. Given the anionic nature of the sulfate substituents, one might expect to observe Me_3_N^+^···^−^O_3_SO interactions in the crystal. Somewhat surprisingly, only a single short Me_3_N^+^···^−^O_3_SO contact (4.352 Å) is observed with distance < 4.4 Å. There are, however, numerous longer Me_3_N^+^···^−^O_3_SO contacts with distances in the 4.4–5.4 Å range which suggests they play a supporting role during crystallization.

### Measurement of the self-association of **C1**

Before proceeding to investigate the molecular recognition properties of **C1** by ITC, we wanted to determine whether **C1** undergoes self-association in phosphate-buffered saline (PBS) which might impinge on guest binding and complicate the determination of **C1**·guest binding constants. For this purpose, we performed dilution experiments monitored by ^1^H NMR spectroscopy. We prepared a series of NMR samples of **C1** in D_2_O (from 4 mM to 125 μM) and monitored the chemical shift of H_a_ (Supporting Information File 1, Figure S14). Over this dilution range, the resonance for H_a_ remains a sharp singlet at 6.94 ppm. Accordingly, we conclude that **C1** remains monomeric at the low concentration (100 μM) typically employed for isothermal titration calorimetry measurements.

### Use of isothermal titration calorimetry to measure the thermodynamic parameters of complexation

**Figure 7:**
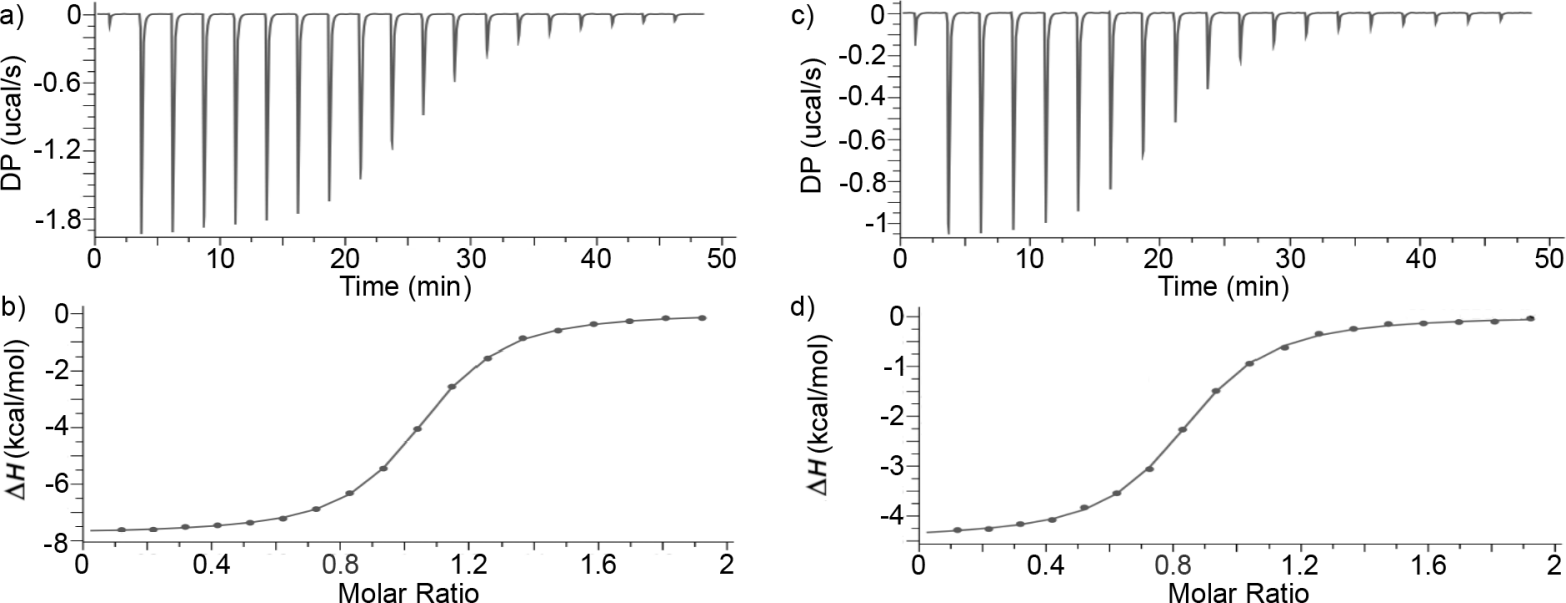
a) Representative plot of DP (μcal s^−1^) versus time from the titration of **C1** (0.1 mM) in the ITC cell with a solution of **CHDA** (1.0 mM) from the ITC syringe. b) Plot of Δ*H* versus the **C1**:**CHDA** molar ratio. The solid line represents the best fit of the data to the single-set-of-sites binding model implemented in the PEAQ ITC data analysis software. The measurements were performed in triplicate and yielded *K*_a_ = (6.49 ± 0.10) × 10^5^ M^−1^ and Δ*H* = −7.82 ± 0.02 kcal mol^−1^. c) Representative plot of DP versus time from the competitive titration of **C1** (0.1 mM) and **CHDA** (0.8 mM) in the cell with a solution of **Me_6_PXDA** (1.0 mM) from the syringe. d) Plot of Δ*H* versus the **C1**:**Me_6_PXDA** molar ratio. The solid line represents the best fit of the data to the competitive binding model implemented in the PEAQ ITC data analysis software. The measurements were performed in triplicate and yielded *K*_a_ = (2.47 ± 0.06) × 10^8^ M^−1^ and Δ*H* = −12.43 ± 0.02 kcal mol^−1^.

Acyclic CB[*n*]-type receptors are known to bind tightly (*K*_a_ > 10^6^ M^−1^) to hydrophobic diammonium ions [42,65,71]. Accordingly, we elected to use isothermal titration calorimetry (ITC) to measure the binding between **C1** or **M1** with the panel of guests. A single ITC run is capable of delivering both the binding constant (*K*_a_, M^−1^) and the enthalpy of complexation (Δ*H*, kcal mol^−1^). Direct ITC titrations are most appropriate for host·guest complexes with *K*_a_ ≤ 10^7^ M^−1^ where Wiseman c-values from 5–500 can be achieved by changing the concentration of host in the ITC cell [76–78]. Figure 7a presents the thermogram recorded when a solution of **C1** (100 μM) in phosphate-buffered saline (PBS) in the ITC cell was titrated with a solution of **CHDA** (1 mM) from the ITC injection syringe. The DP versus time data in Figure 6a was integrated and then plotted as Δ*H* versus molar ratio in Figure 7b. The Δ*H* versus molar ratio data was then fitted to the single-set-of-sites binding model in the PEAQ ITC data analysis software which delivered *K*_a_ = (6.49 ± 0.10) × 10^5^ M^−1^ and Δ*H* = −7.82 ± 0.02 kcal mol^−1^ for the **C1**·**CHDA** complex (Table 1). All ITC experiments were performed in triplicate and the reported values represent the mean ± standard deviation. For stronger complexes, where the Wiseman c-value cannot be adjusted into the ideal range by reducing the host concentration in the ITC cell due to the insufficient heat evolved, competitive ITC titrations must be used. In competitive ITC titrations a solution of the host and an excess of a weak binding competitive guest in the ITC cell is titrated with a solution of the tighter binding guest from the ITC injection syringe [78]. The analysis of competitive titrations requires that the *K*_a_ and Δ*H* values for the host·competitor complexes have been previously determined and used as known inputs to the competitive binding model in the PEAQ data analysis software. To maximize the heat evolved in the competitive ITC titrations, the host·competitor and host·tight guest complexes should have very different Δ*H* values. Figure 7c shows the competitive ITC titration of a mixture of **C1** (0.1 mM) and **CHDA** (0.8 mM) in the ITC cell with a solution of **Me_6_PXDA** (1.0 mM) from the syringe. The DP versus time plot was integrated and a plot of Δ*H* versus molar ratio was created (Figure 7d) and fitted to the competitive binding model in the PEAQ ITC data analysis software to determine *K*_a_ = (2.47 ± 0.06) × 10^8^ M^−1^ and Δ*H* = −12.43 ± 0.02 kcal mol^−1^ for the **C1**·**Me_6_PXDA** complex. The *K*_a_ and Δ*H* values for the remaining **C1**·guest and **M1**·guest complexes were determined by analogous direct or competitive ITC titrations (Table 1 and Supporting Information File 1).

**Table 1:** Thermodynamic parameters (*K*_a_ (M^−1^), ∆*H*° (kcal/mol) determined for the **C1**·guest, **M1**·guest and **M0**·guest complexes by ITC. Conditions: 298.0 K, phosphate-buffered saline, pH 7.4.

Guest	**C1**		**M1**	
	*K*_a_	∆*H*°	*K*_a_ (M^−1^)	∆*H*°

**Me** **_6_** **PDA**	(3.40 ± 0.09) × 10^7d^	−13.47 ± 0.03	(1.31 ± 0.05) × 10^6a^	−5.98 ± 0.03
**Me** **_6_** **HDA**	(6.54 ± 0.59) × 10^7b^	−10.13 ± 0.02	(2.95 ± 0.12) × 10^6a^	−5.27 ± 0.02
**PXDA**	(1.44 ± 0.03) × 10^8c^	−10.07 ± 0.01	(3.42 ± 0.05) × 10^7c^	−5.67 ± 0.01
**Me** **_6_** **PXDA**	(2.47 ± 0.06) × 10^8b^	−12.43 ± 0.02	(7.52 ± 0.18) × 10^7b^	−8.64 ± 0.02
**CHDA**	(6.49 ± 0.10) × 10^5a^	−7.82 ± 0.02	(2.79 ± 0.07) × 10^5a^	−4.38 ± 0.02
**Me** **_6_** **CHDA**	(1.75 ± 0.06) × 10^6a^	−7.83 ± 0.03	(1.20 ± 0.04) × 10^6a^	−7.44 ± 0.03
**AdA**	(2.41 ± 0.04) × 10^6a^	−7.54 ± 0.03	(1.99 ± 0.06) × 10^6a^	−4.11 ± 0.03
**Me** **_3_** **AdA**	(2.31 ± 0.07) × 10^6a^	−11.00 ± 0.04	(2.09 ± 0.07) × 10^6a^	−7.42 ± 0.02

^a^Measured by direct ITC titration of host (100 μM) in the cell with guest (1 mM) in the syringe. ^b^Measured by ITC competition assay using **CHDA** (0.8 mM) as competitor included in the cell. ^c^Measured by ITC competition assay using **CHDA** (0.5 mM) as competitor included in the cell. ^d^Measured by ITC competition assay using **CHDA** (0.1 mM) as competitor included in the cell.

### Comparison of the thermodynamic parameters for **C1**·guest and **M1**·guest complexation

Overall, **C1** is the more potent host with *K*_a_ values ranging from 2.41 × 10^5^ (**AdA**) to 2.49 × 10^8^ M^−1^ (**Me_6_PXDA**) relative to **M1** whose *K*_a_ values range from 1.99 × 10^5^ (**AdA**) to 7.52 × 10^7^ M^−1^ (**Me_6_PXDA**). Similarly, the enthalpic contributions to binding are more favorable for **C1** with Δ*H* values ranging from −7.54 (**AdA**) to −13.47 kcal mol^−1^ (**Me_6_PDA**) than for **M1** with Δ*H* values ranging from −4.11 (**AdA**) to −8.64 kcal mol^−1^ (**Me_6_PXDA**). The more favorable enthalpic contributions to binding is likely due to stronger electrostatic interactions between the guest and the sulfate ionic groups. For both **C1** and **M1**, the **Me_6_HDA** and **Me_6_PXDA** are the strongest binding guests whereas the cyclohexane and adamantane-based guests with a larger cross-sectional area bind 10–100-fold more weakly. The ratio of the binding constants of a common guest to **C1** versus **M1** is as follows: **Me_6_PDA** (26.0), **Me_6_HDA** (22.2), **PXDA** (4.2), **Me_6_PXDA** (3.3), **CHDA** (2.3), **Me_6_CHDA** (1.3), **AdA** (1.2), **Me_3_AdA** (1.1). The **C1** host is both a tighter and more selective host for the narrower guests than **M1**. We can also tease out the effect of chain length by a comparison of **Me_6_PDA** with **Me_6_HDA**. We find that the longer and more hydrophobic **Me_6_HDA** guest binds 1.92-fold stronger to **C1**; similarly, **Me_6_HDA** binds 2.25-fold stronger to **M1**. These differences are likely due to the increased hydrophobicity of the additional CH_2_ group. Finally, we can compare the binding of the primary ammonium versus the corresponding quaternary ammonium ion guest toward **C1** and separately **M1**. We find that **C1** binds the quaternary ammoniums somewhat stronger: **Me_6_PXDA** vs **PXDA** (1.72-fold), **Me_6_CHDA** vs **CHDA** (2.42-fold), **Me_3_AdA** vs **AdA** (4.59-fold). A similar trend holds for **M1**: **Me_6_PXDA** vs **PXDA** (2.20-fold), **Me_6_CHDA** vs **CHDA** (4.30-fold), **Me_3_AdA** vs **AdA** (10.50-fold).

## Conclusion

In summary, we have designed, synthesized, and characterized a new acyclic CB[*n*]-type receptor **C1** that bears sulfate ionic groups and compared its properties with **M1** which features sulfonate ionic groups. We find that **C1** is much less soluble (4 mM) than **M1** (346 mM) in water. Host **C1** does not undergo self-association in PBS buffer according to ^1^H NMR dilution experiments. Analysis of complexation-induced changes in chemical shifts establish that the hydrophobic regions of the guests bind within the anisotropic shielding cavity of **C1** whereas the ionic groups reside closer to the ureidyl carbonyl portals of **C1**. Direct and competitive ITC titrations were used to measure the thermodynamic parameters of binding for **C1**·guest and **M1**·guest complexes in PBS solution. Overall, we find that **C1** – with its sulfate ionic groups – binds tighter than **M1** toward each member of the guest panel with largest differences observed for the narrowest **Me_6_PDA** (26-fold) and **Me_6_HDA** (22.2-fold) guests. Similarly, we find that **C1** binds the quaternary ammonium stronger than the corresponding primary ammonium ion guest by 1.72 to 9.59-fold. In conclusion, we find that **C1** displays somewhat enhanced molecular recognition properties than **M1** but possesses less desirable aqueous solubility properties.

## Experimental

### General experimental details

All chemicals were purchased from commercial suppliers and were used without further purification. Guest molecules were available from previous studies [65,71]. Compounds **TetW1_OAc_** and **TetW1** were prepared according to the literature procedures with slight modifications [70]. NMR spectra were recorded using commercial spectrometers operating at 600 or 400 MHz for ^1^H and 150 or 100 MHz for ^13^C. Melting points were measured on a Meltemp apparatus in open capillary tubes and are uncorrected. IR spectra were measured on a Thermo Nicolet NEXUS 670 FT/IR spectrometer by attenuated total reflectance (ATR) and are reported in cm^−1^. Mass spectrometry was performed using a JEOL AccuTOF electrospray instrument. ITC data was collected on a Malvern Microcal PEAQ-ITC instrument with a cell volume of 200 µL and an injection syringe with a capacity of 40 μL. For ITC experiments, the host and guest solutions were prepared in a 20 mM phosphate-buffered water (pH 7.4). The sample cell was filled (200 μL) with the host solution and the guest solution was titrated (first injection = 0.4 μL, subsequent 18 injections = 2 μL) into the cell. All ITC experiments were analyzed using the MicroCal PEAQ-ITC data analysis software.

### Compound **C1**

A mixture of **TetW1** (0.430 g, 0.376 mmol) and pyridine sulfur trioxide (1.1838 g, 7.437 mmol) was dissolved in dry pyridine (57 mL). The resulting mixture was heated at 90 °C under N_2_ for 18 h and then cooled to rt. The precipitate was collected by first decanting some of the solvent and then the remaining mixture was transferred to a 50 mL centrifuge tube and centrifuged (7200 rpm, 5 min). The supernatant was carefully poured off. Next, the crude solid was dissolved in 1 M NaOH (25 mL) which results in a yellow and then red solution. Afterwards, EtOH (144 mL) was added which gave a white precipitate that was collected by centrifugation (7200 rpm, 10 min). The crude solid was analyzed by ^1^H NMR which showed residual pyridine. The crude solid was subsequently dissolved in water (150 mL) and re-precipitated by the addition of EtOH (144 mL) followed by centrifugation (7200 rpm, 5 min) to obtain a white solid. The solid was dried overnight under high vacuum to yield **C1** as a white solid (0.3444 g, 68% yield). Mp > 300 °C; IR (ATR, cm^−1^): 3456 (w), 1720 (m), 1472 (m), 1378 (w), 1226 (m), 1101 (s), 1023 (m), 972 (w), 790 (w); ^1^H NMR (400 MHz, D_2_O) 6.71 (s, 4H), 5.67 (d, *J* = 15.4 Hz, 2H), 5.57 (d, *J* = 15.8 Hz, 4H), 5.46 (d, *J* = 8.9 Hz, 2H), 5.42 (d, *J* = 8.9 Hz, 2H), 5.41 (d, *J* = 16.3 Hz, 4H), 4.45–4.40 (m, 4H), 4.35–4.30 (m, 4H), 4.27 (d, *J* = 15.8 Hz, 2 × 4H), 4.25–4.18 (m, 4H), 4.18–4.13 (m, 4H), 4.12 (d, *J* = 15.4 Hz, 2H), 1.78 (s, 6H), 1.75 (s, 6H); ^13^C NMR (100 MHz, D_2_O/acetone-*d*_6_ 6:1 (v:v)) 156.7, 155.9, 150.5, 129.3, 116.1, 78.6, 77.4, 71.2, 69.5, 67.4, 52.5, 48.4, 35.0, 16.3, 15.4 ppm; ESIMS (*m*/*z*): 751.13 ([M − 2Na]^2−^), calcd for [C_50_H_56_N_16_Na_2_S_4_O_28_]^2−^, 751.1064.

## Supporting Information

The X-ray crystal structure of **C1** is deposited with the Cambridge Crystallographic Data Centre (CCDC 2411723).

File 1 Synthesis and characterization of compounds, solubility determination, ^1^H NMR dilution experiments, ^1^H NMR and ITC binding studies.

## Data Availability

Data generated and analyzed during this study is openly available in Digital Repository at the University of Maryland (UMD DRUM) at https://doi.org/10.13016/4gal-sini.
