# Supplementary material for: Acyclic cucurbit[n]uril bearing alkyl sulfate ionic groups
Source: Beilstein J Org Chem. 2025 Apr 3;21:717–26. doi: 10.3762/bjoc.21.55 (PMC11973588; doi:10.3762/bjoc.21.55)
Supplement: File 1 — Synthesis and characterization of compounds, solubility determination, 1H NMR dilution experiments, 1H NMR and ITC binding studies. [file Beilstein_J_Org_Chem-21-717-s001.pdf]

BEILSTEIN JOURNAL OF ORGANIC CHEMISTRY

Supporting Information

for

Acyclic cucurbit[*n*]uril bearing alkyl sulfate ionic groups

Christian Akakpo, Peter Y. Zavalij and Lyle Isaacs

Beilstein J. Org. Chem. **2025,** *21,* 717–726. doi:10.3762/bjoc.21.55

Synthesis and characterization of compounds, solubility determination, ^{1}H NMR dilution experiments, ^{1}H NMR and ITC binding studies

License and Terms: This is a supporting information file under the terms of the Creative Commons Attribution License (https://creativecommons.org/licenses/by/4.0). Please note that the reuse, redistribution and reproduction in particular requires that the author(s) and source are credited and that individual graphics may be subject to special legal provisions.
The license is subject to the *Beilstein Journal of Organic Chemistry* terms and conditions: (https://www.beilstein-journals.org/bjoc/terms)

Table of contents Pages

General experimental details S2

Synthesis and characterization of compounds S3

Additional references S4

NMR spectra of **TetW1$_{\mathbf{OAc}}$**, **TetW1**, and **C1** S5

Determination of the solubility of **C1** S9

^{1}H NMR stack plots for complexes of **C1** with the guests S11

Dilution experiment for **C1** S19

Isothermal titration calorimetry experiments for **C1** S20

Isothermal titration calorimetry experiments for **M1** S28

General experimental details. All reagents and chemicals were purchased from commercial suppliers and were used without further purification. All guest molecules were purchased from commercial suppliers. The synthesis of **TetBCE**, **TetW1$_{\mathbf{OAc}}$**, **TetW1**, and **M1** has been reported previously in the literature.[1,2] ^{1}H NMR spectra were measured at 400 or 600 MHz and ^{13}C NMR spectra were measured at 100 or 150 MHz using the indicated solvents. Melting points were measured on a Meltemp apparatus in open capillary tubes and are uncorrected. IR spectra were measured on a Thermo Nicolet NEXUS 670 FT/IR spectrometer by attenuated total reflectance (ATR) and are reported in cm^{-1}. Mass spectrometry was performed using a JEOL AccuTOF electrospray Instrument. ITC data was collected on a Malvern Microcal PEAQ-ITC instrument with a 200 μL cell volume and a 40 μL injection syringe capacity. For ITC experiments, the host and guest solutions were prepared in phosphate-buffered saline (pH 7.4). PBS was prepared by dissolving the commercially available tablets (MP Biomedical, catalog #2810305) in HPLC water. The sample cell was filled (200 μL) with the host solution and the guest solution was titrated (first injection = 0.4 μL, subsequent 18 injections = 2 μL) into the cell. All ITC experiments were analyzed using the PEAQ-ITC data analysis software provided by the manufacturer.

Synthesis and characterization of compounds

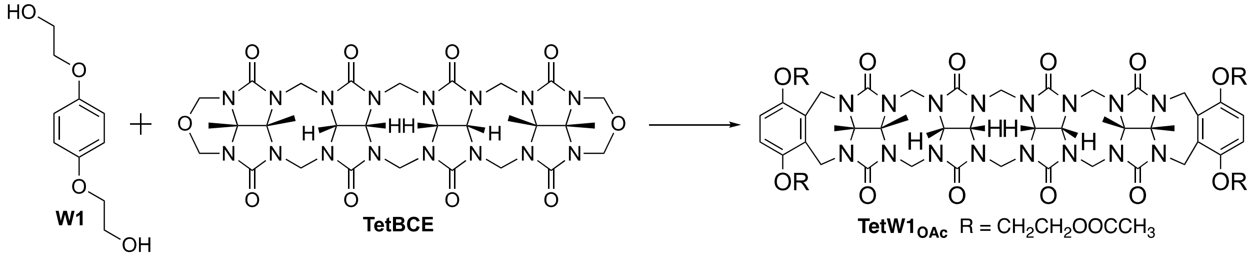

Compound TetW1OAc. In a manner similar to the literature procedure,[3] a mixture of **W1** (1.000 g, 1.28 mmol) and **TetBCE** (1.021 g, 5.12 mmol) was dissolved in a mixture of TFA and Ac_2O 1:1 (v:v, 10 mL) and then heated at 70 °C for 3.5 h. At that point, the hot reaction mixture was poured into MeOH (150 mL) which resulted in the formation of a white precipitate. The mixture was transferred into 50 mL centrifuge tubes and then centrifuged (7200 rpm, 10 min). The supernatant was carefully poured off. The resulting solid was resuspended in acetone (100 mL), sonicated for 10 min, and then centrifuged (7200 rpm, 10 min). The supernatant was poured off carefully and then the solid was resuspended in water (100 mL), sonicated for 10 min, and then centrifuged (7200 rpm, 10 min). The supernatant was carefully poured off and then the solid was dried under high vacuum overnight, yielding **TetW1OAc** (80.6 mg, 71% yield) as a white powder. The ^1H NMR spectrum matches that reported in the literature.[3]

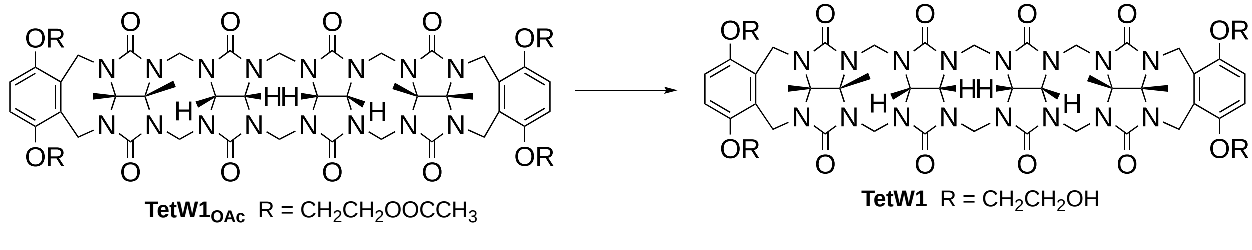

Compound TetW1. In a manner similar to the literature procedure,[3] **TetW1OAc** (0.400 g, 0.305 mmol) was added into an aqueous solution of LiOH (2.5 M, 7.5 mL). The mixture was stirred at 50 °C for 0.5 h. The heterogenous reaction mixture was transferred to a centrifuge tube

and centrifuged (7200 rpm, 10 min). The supernatant was poured off to give a crude solid. The crude solid was dissolved in water and then 0.1 M HCl was added portionwise until the solution had pH 7.0. The precipitate was isolated by centrifugation (7200 rpm, 10 min) to give a crude solid after pouring off the supernatant. The crude solid was slurried in EtOH (30 mL), sonicated for 15 min, and then centrifuged (7200 rpm, 10 min). The supernatant was poured off to give a crude white solid. The crude solid was then slurried in water (30 mL), sonicated for 15 min, and then centrifuged (7200 rpm, 10 min). The supernatant was poured off to give a white solid. The white solid was dried under high vacuum overnight, yielding **TetW1** as a white solid (234 mg, 69% yield). The ^{1}H NMR spectrum matches that reported in the literature.[3]

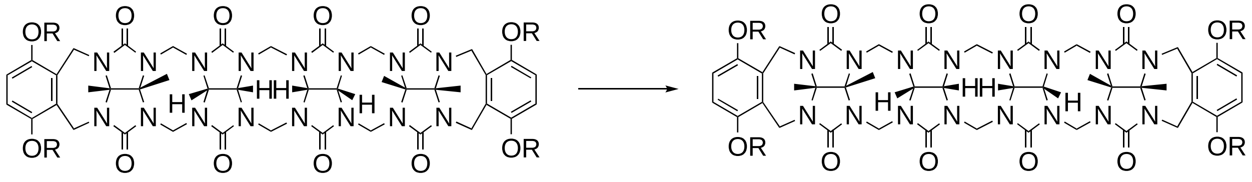

TetW1 R = CH_2CH_2OH **C1** R = $CH_2CH_2OSO_3Na$

Compound C1. The synthesis and characterization data for this compound is described in the main article.

References

(1) Zhang, B.; Isaacs, L. *J. Med. Chem.* **2014**, *57*, 9554–9563.

(2) Brockett, A. T.; Deng, C.; Shuster, M.; Perera, S.; DiMaggio, D.; Cheng, M.; Murkli, S.; Briken, V.; Roesch, M. R.; Isaacs, L. *Chem. Eur. J.* **2021**, *27*, 17476–17486.

(3) Zhang, B.; Zavalij, P. Y.; Isaacs, L. *Org. Biomol. Chem.* **2014**, *12*, 2413–2422.

(4) Yao, Y.; Xue, M.; Chi, X.; Ma, Y.; He, J.; Abliz, Z.; Huang, F. *Chem. Commun.* **2012**, *48*, 6505.

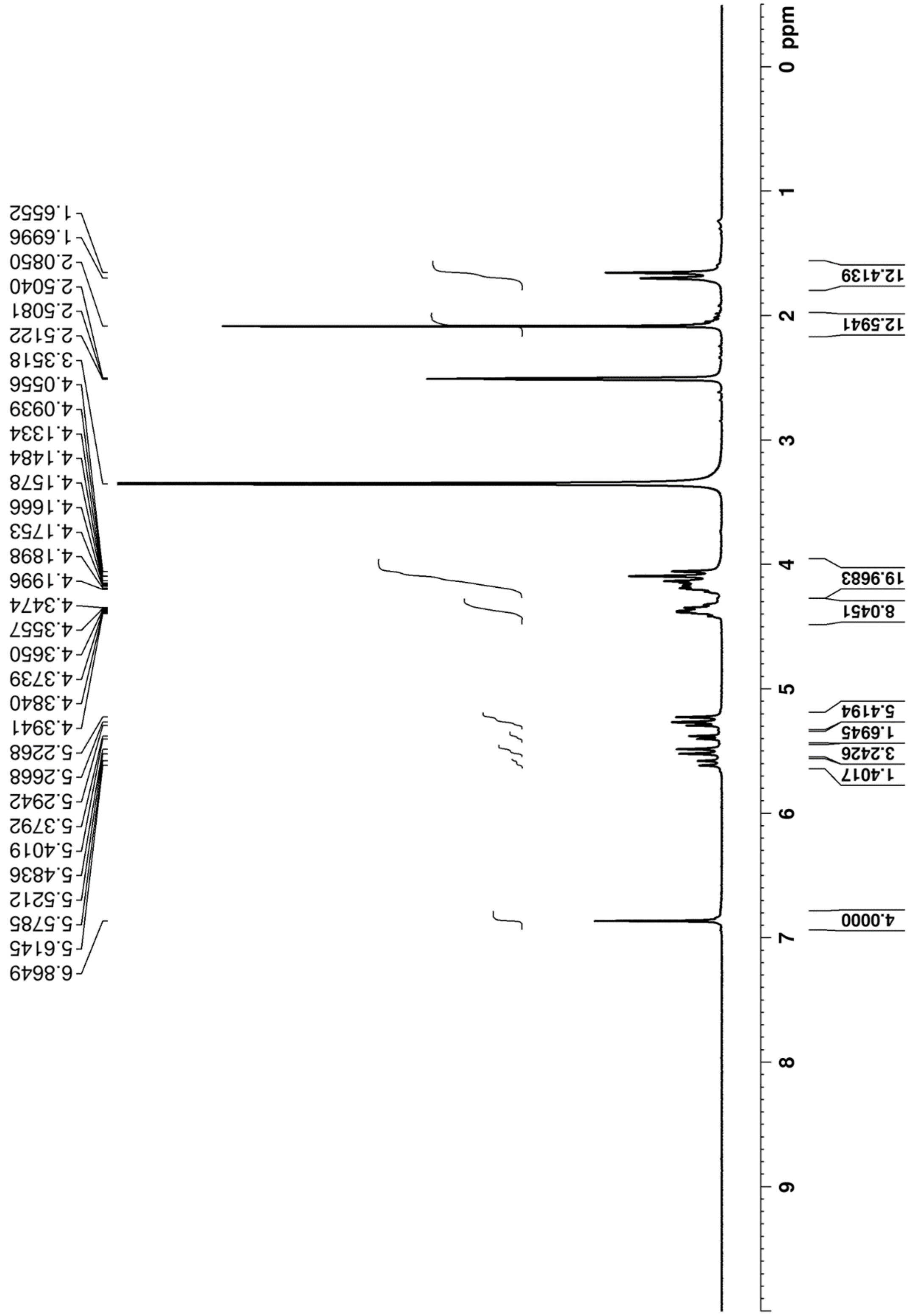

Figure S1. ^{1}H NMR recorded (400 MHz, DMSO-d_6) for **TetW1$_{\mathbf{OAc}}$**.

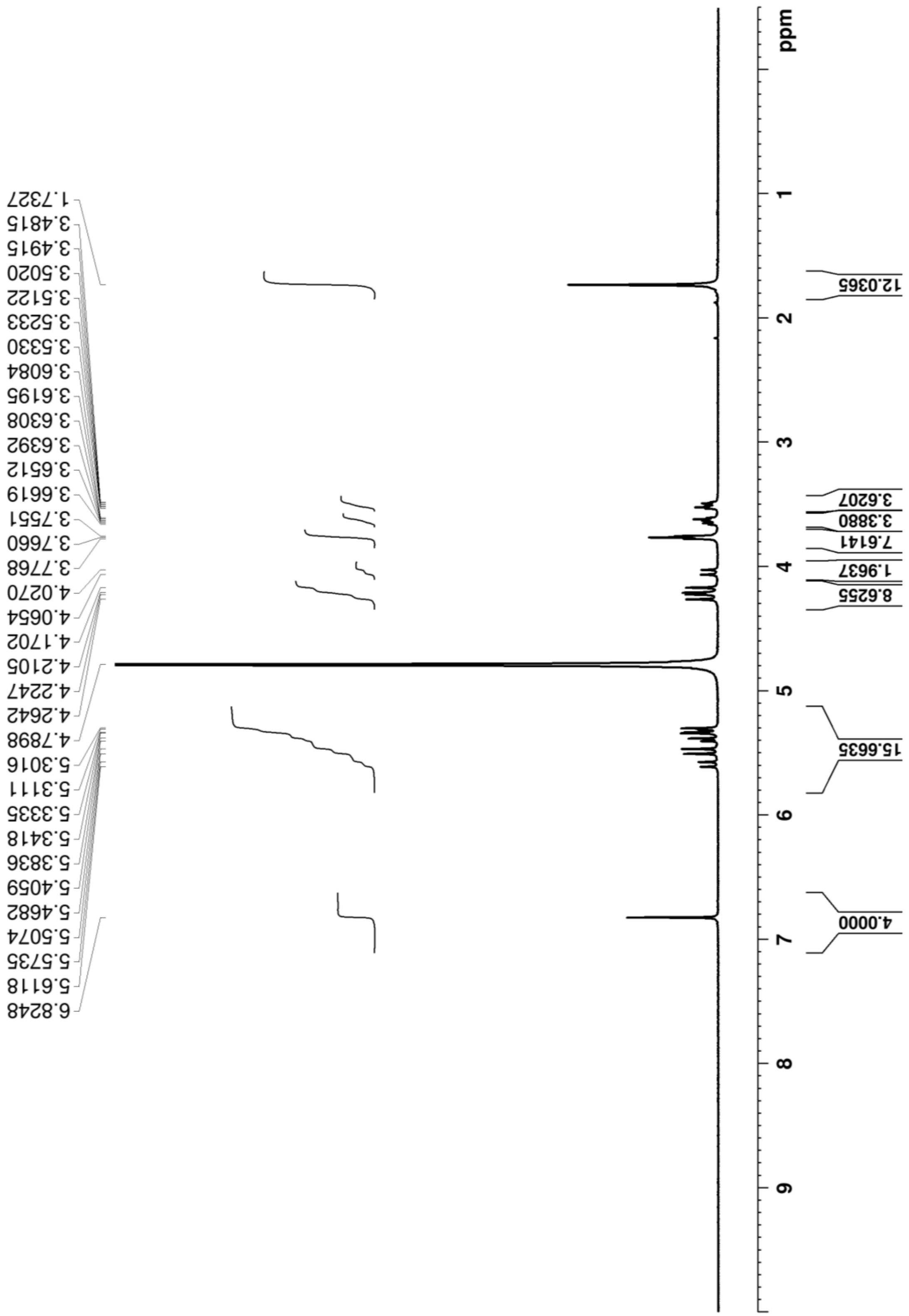

Figure S2. ^{1}H NMR recorded (400 MHz, D_2O) for **TetW1**.

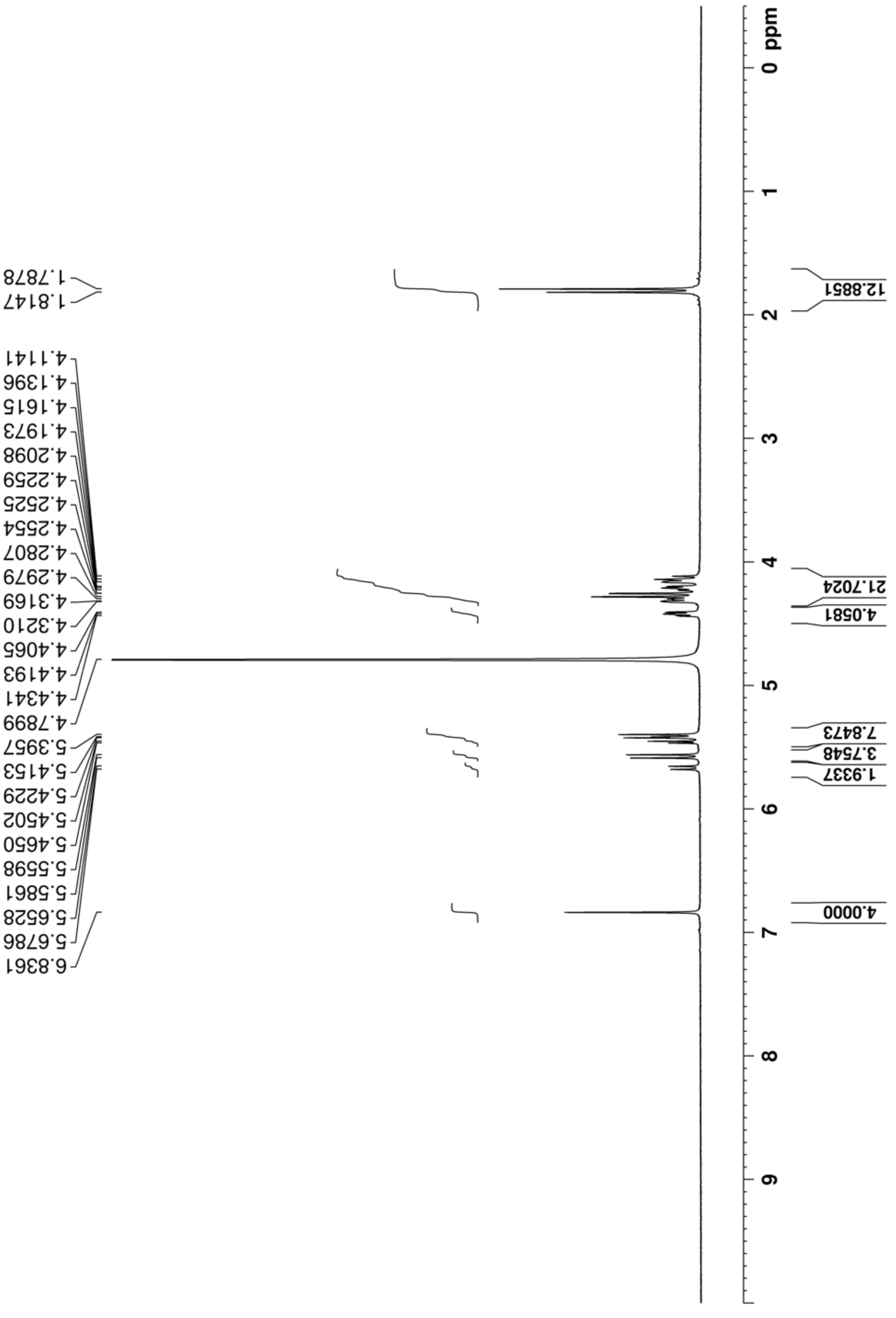

Figure S3. [1] H NMR recorded (600 MHz, D_2O) for **C1**.

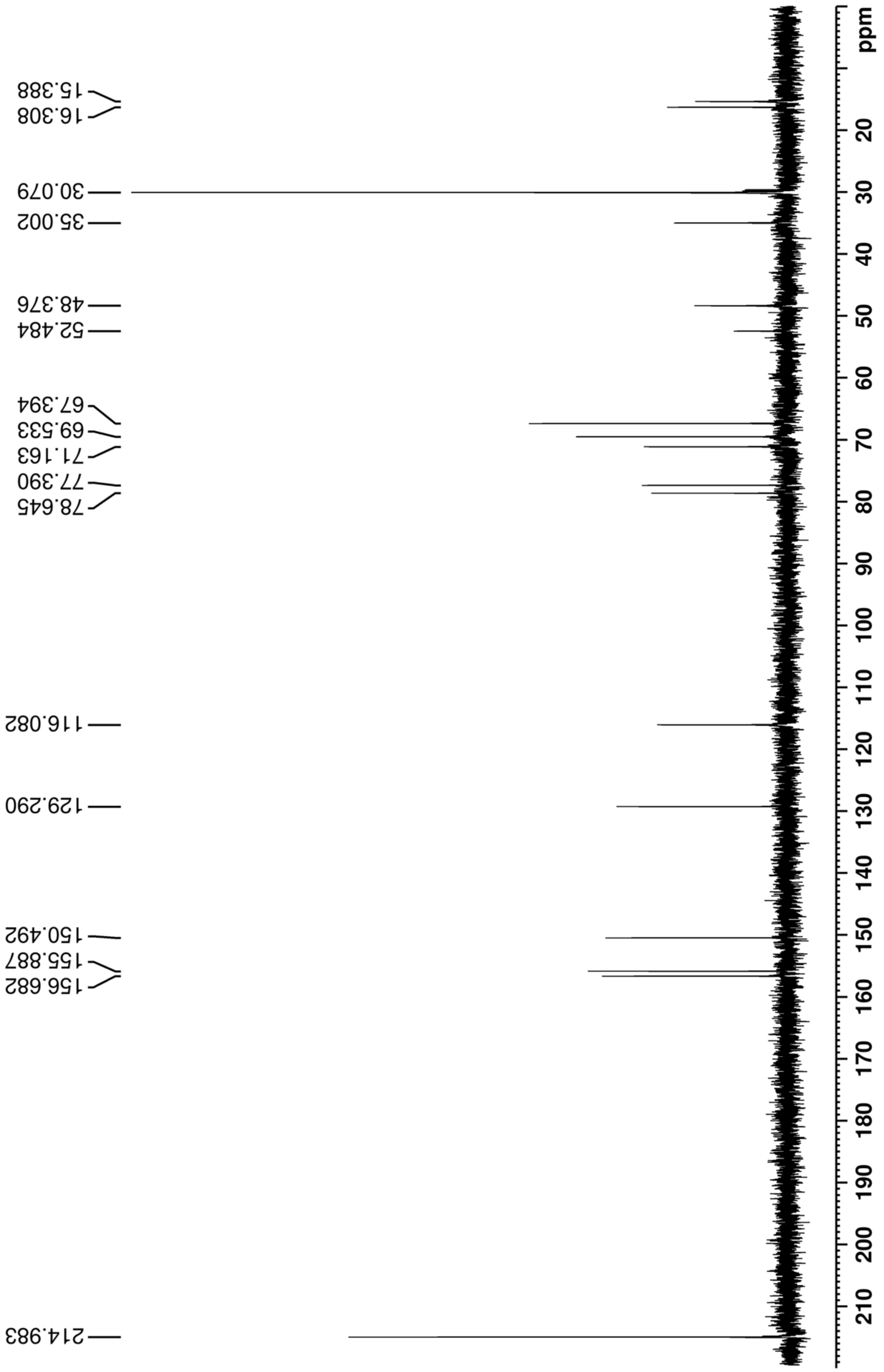

Figure S4. ^{13}C NMR recorded (150 MHz, D_2O/acetone 0.6:0.1) for **C1**.

Determination of the solubility of C1

The solubility of **C1** in water was determined by adding an excess of **C1** (20.4 mg) to D_2O (1.5 mL) in a vial. The resulting suspension was stirred at room temperature overnight and then transferred to a centrifuge tube and centrifuged (4400 rpm, 10 min). An aliquot of the supernatant (100 µL) and an aliquot of dimethyl malonic acid (44.6 mM, 13.6 µL) as internal standard of known concentration was mixed in a vial containing D_2O (486.4 µL) and then transferred to an NMR tube for analysis. The concentration of **C1** host was calculated as 3.97 mM by the comparison of the integral of the aromatic C–H resonance for **C1** with that of the CH_3 resonance for dimethyl malonic acid using the ^1H NMR spectrum shown below (Figure S5) which was collected with a delay time of 20 seconds (D1 = 20 s).

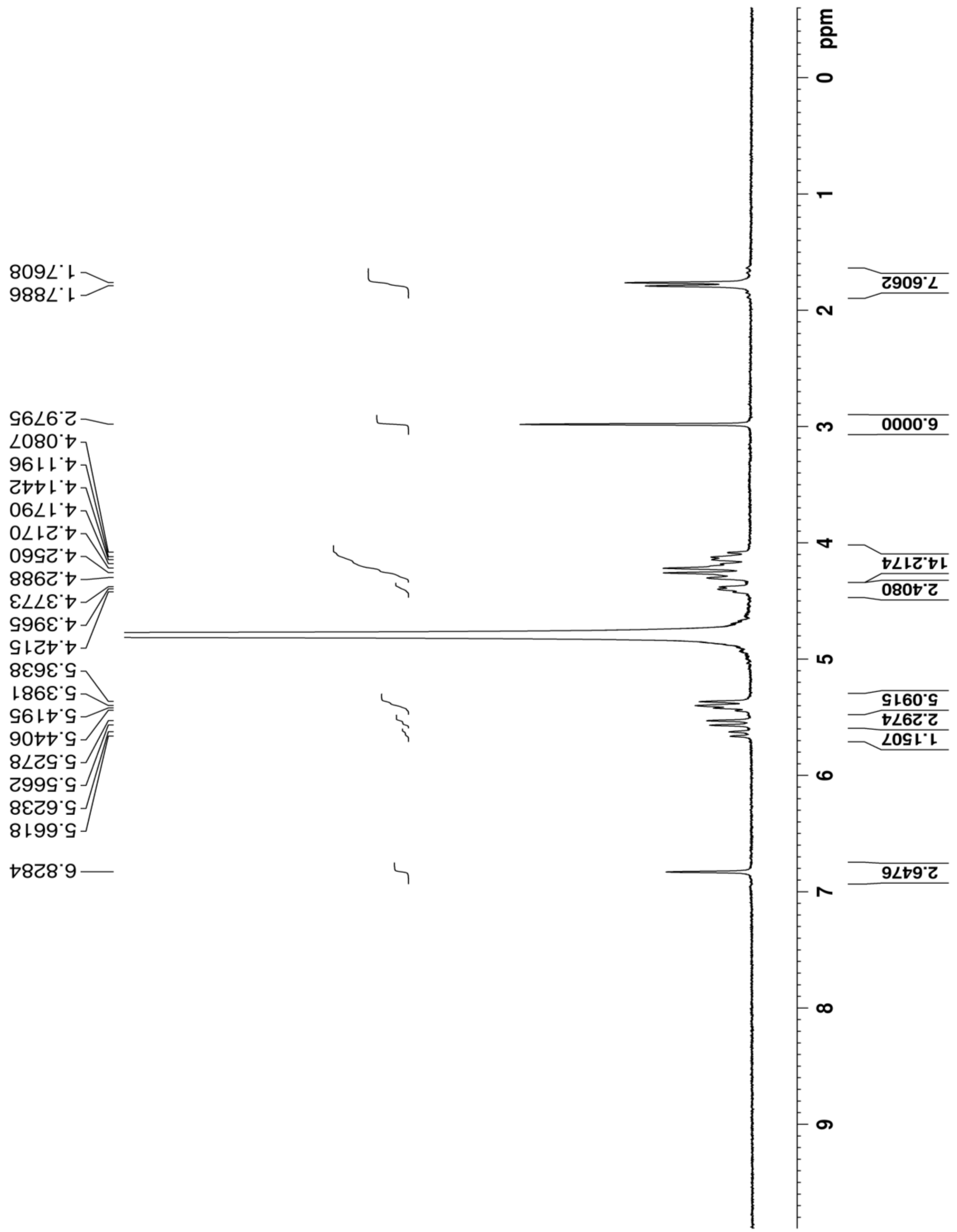

Figure S5. ^{1}H NMR spectra of **C1** versus dimethyl malonic acid as the internal standard (Conditions: 400 MHz, D_2O, rt, D1 = 20 s) used to calculate the solubility of **C1**.

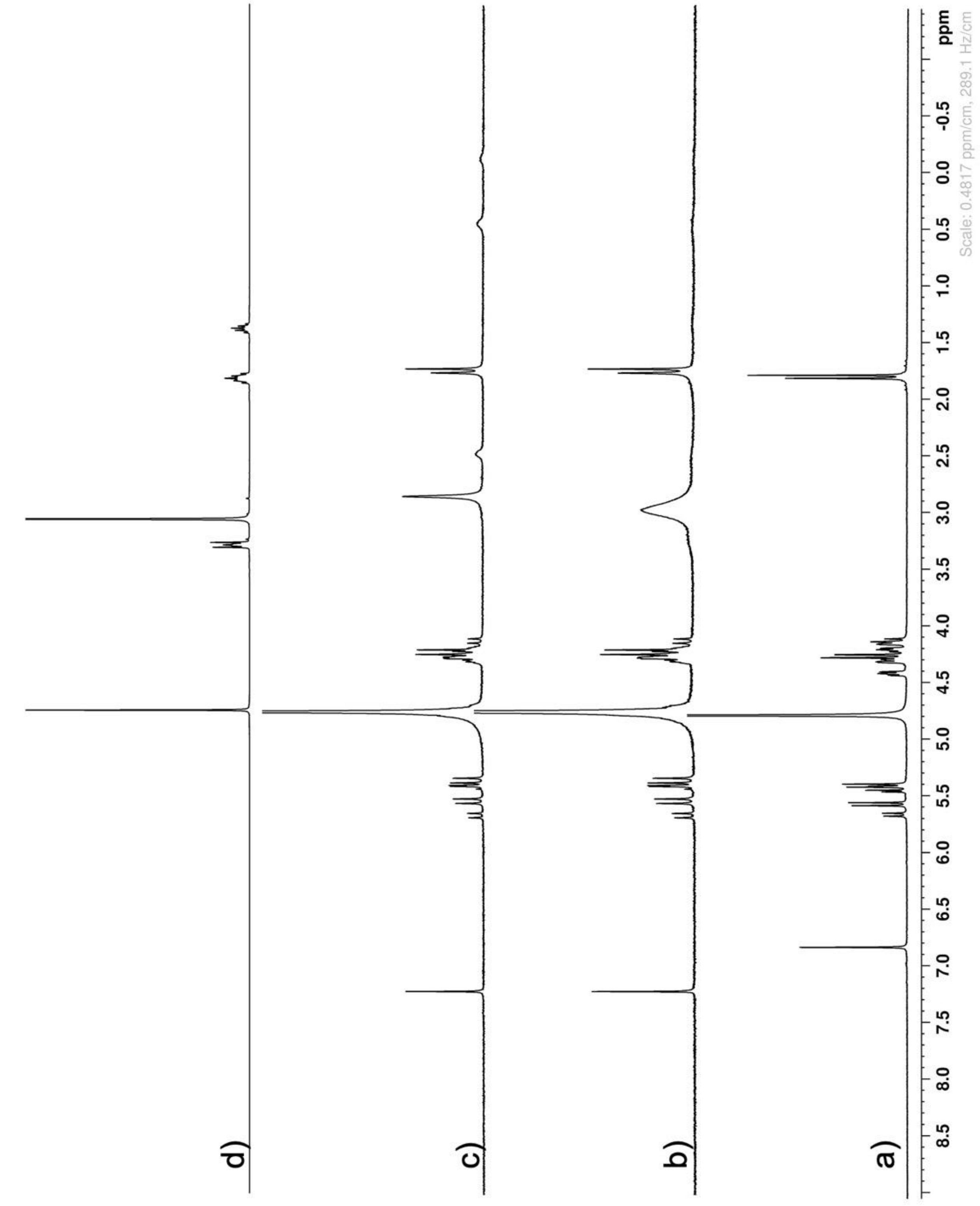

Figure S6. ^{1}H NMR spectra (400 MHz, D_2O, rt) recorded for: a) **C1** (0.5 mM), b) **C1** (0.5 mM) and **Me_6PDA** (0.5 mM), c) **C1** (0.5 mM) and **Me_6PDA** (1.0 mM), and d) **Me_6PDA** (0.5 mM).

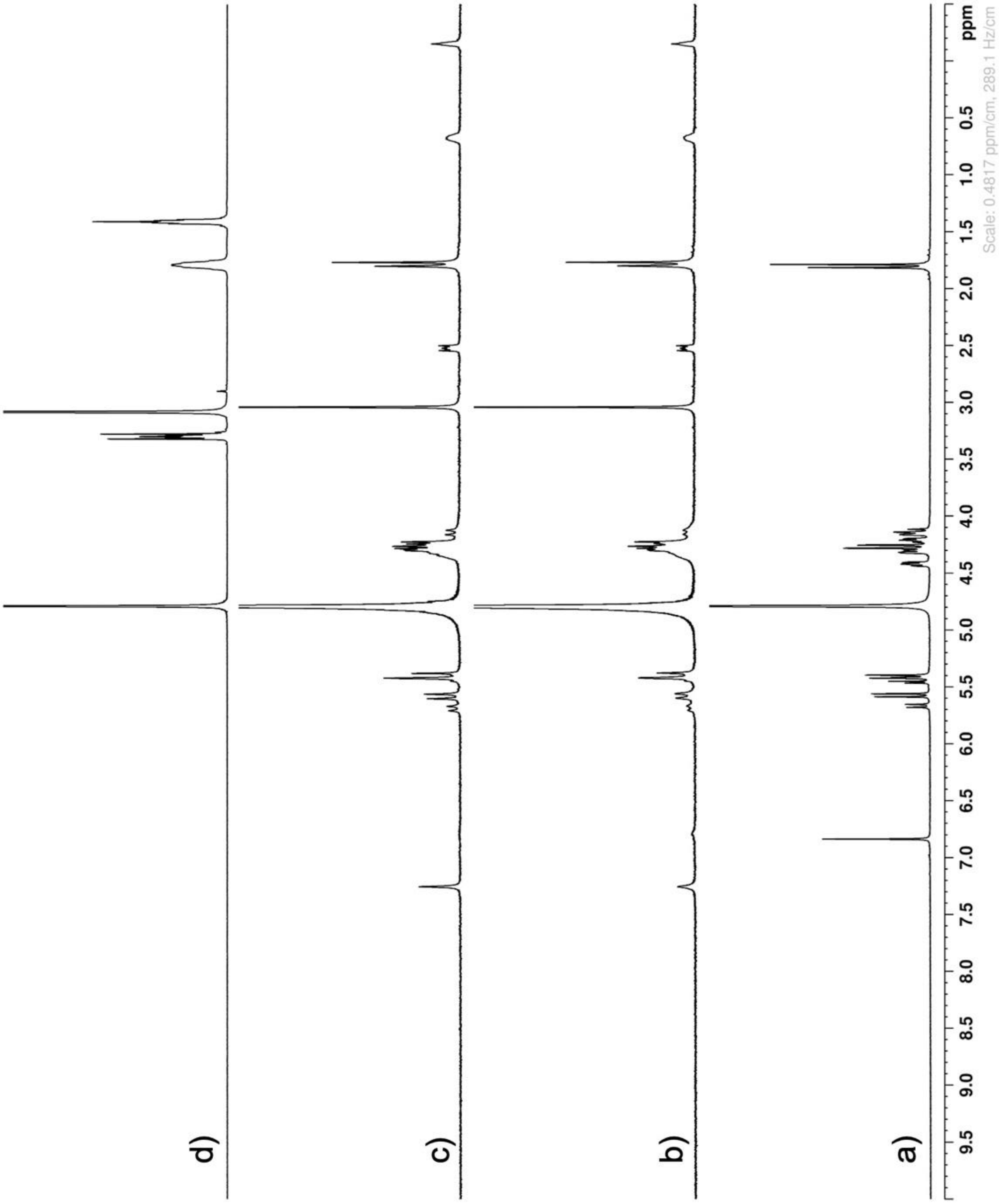

Figure S7. ^{1}H NMR spectra (400 MHz, D_2O, rt) recorded for: a) **C1** (0.5 mM), b) **C1** (0.5 mM) and **Me$_6$HDA** (0.5 mM), c) **C1** (0.5 mM) and **Me$_6$HDA** (1.0 mM), and d) **Me$_6$HDA** (0.5 mM).

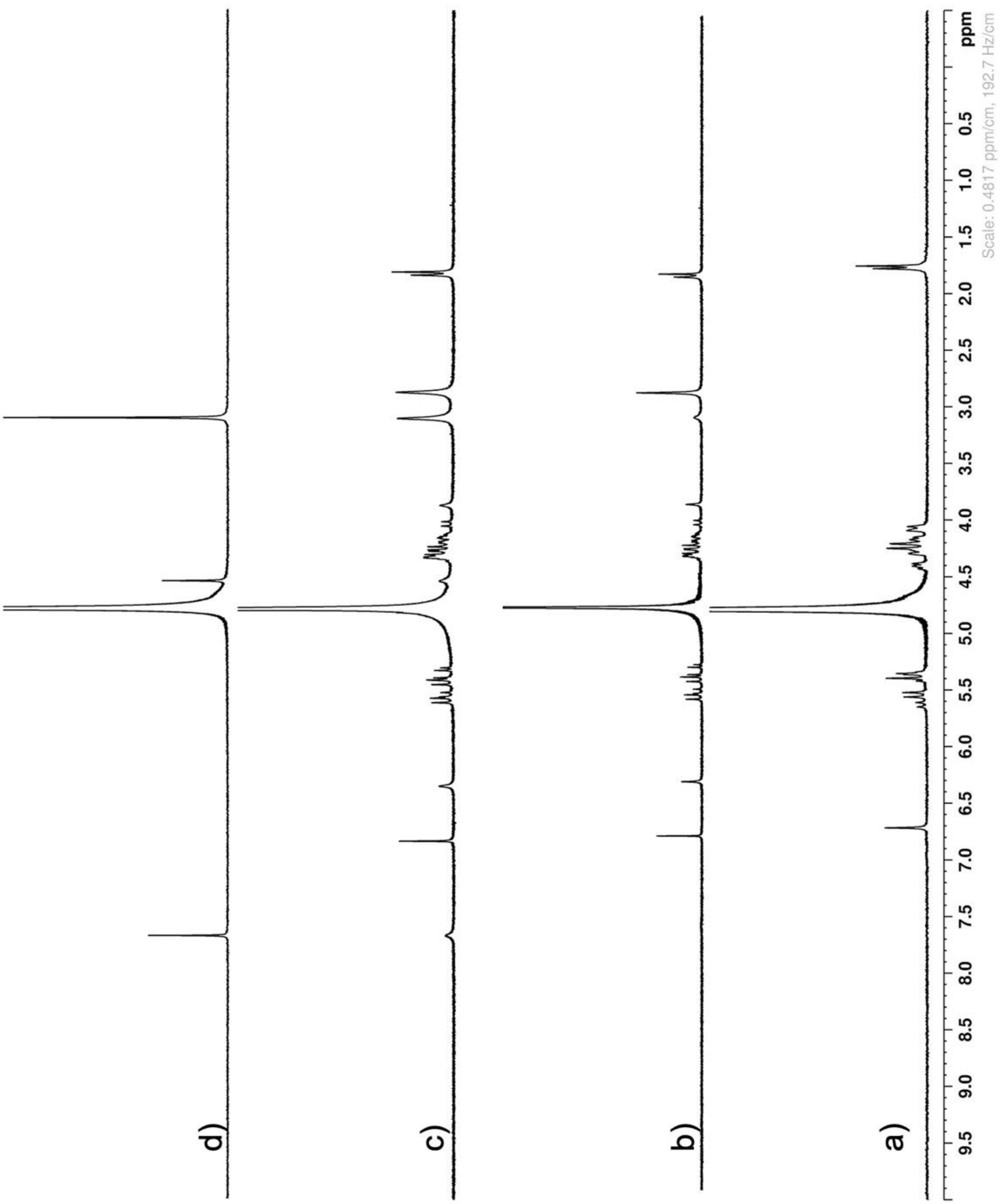

Figure S8. ^{1}H NMR spectra (400 MHz, D_2O, rt) recorded for: a) **C1** (0.5 mM), b) **C1** (0.5 mM) and **PXDA** (0.5 mM), c) **C1** (0.5 mM) and **PXDA** (1.0 mM), and d) **PXDA** (0.5 mM).

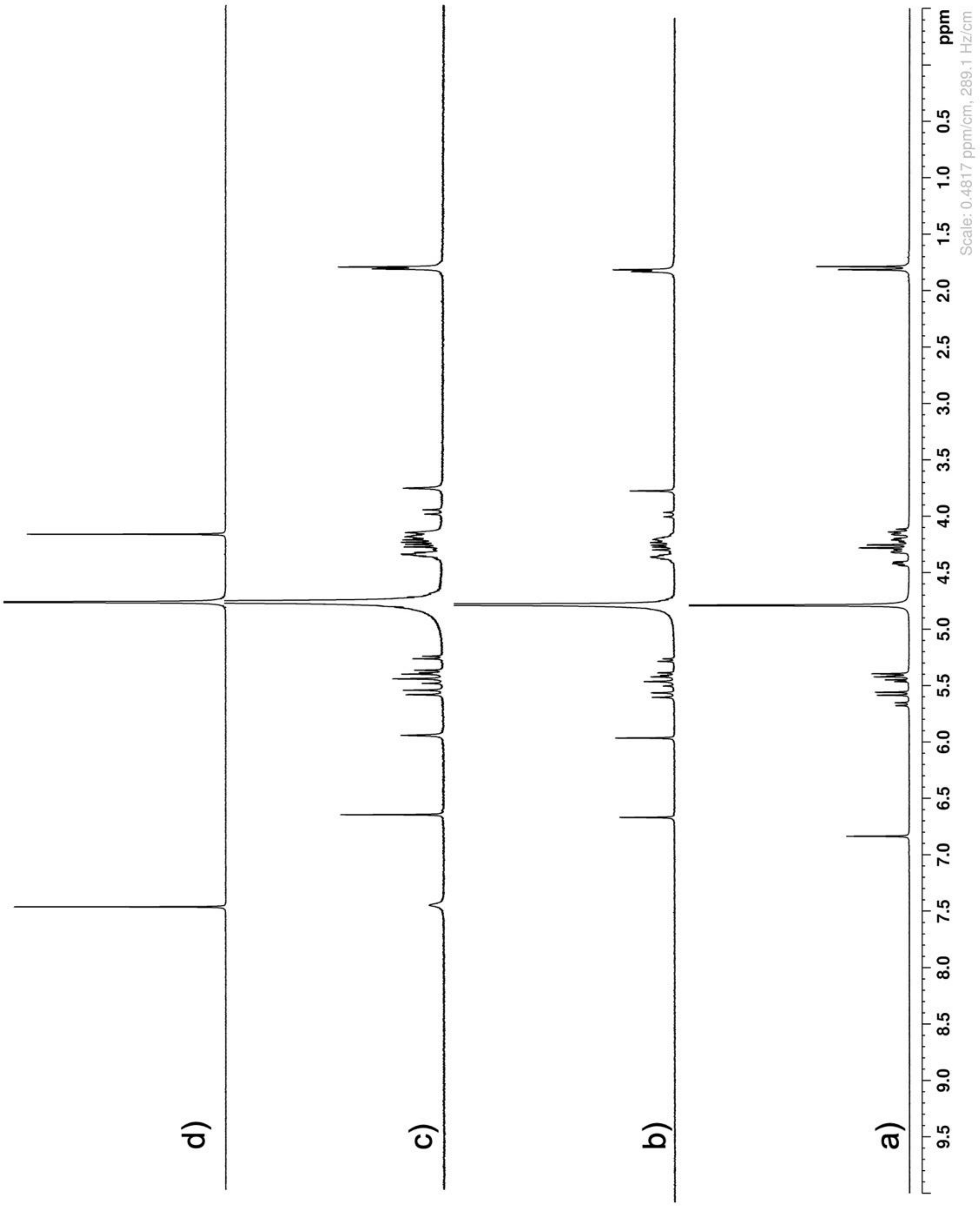

Figure S9. ^{1}H NMR spectra (400 MHz, D_2O, rt) recorded for: a) **C1** (0.5 mM), b) **C1** (0.5 mM) and **Me$_6$PXDA** (0.5 mM), c) **C1** (0.5 mM) and **Me$_6$PXDA** (1.0 mM), and d) **Me$_6$PXDA** (0.5 mM).

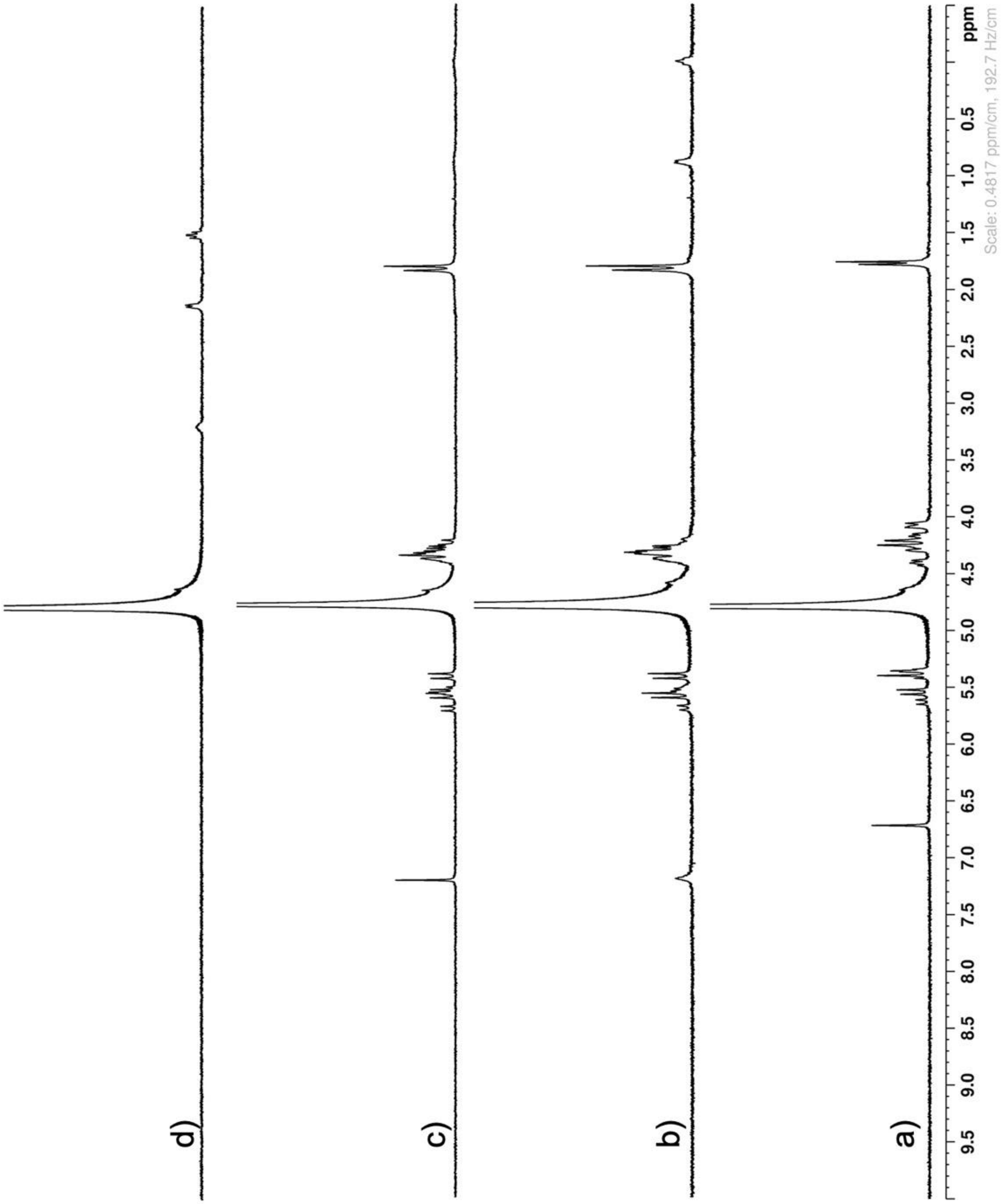

Figure S10. ^{1}H NMR spectra (400 MHz, D_2O, rt) recorded for a) **C1** (0.5 mM), b) **C1** (0.5 mM) and **CHDA** (0.5 mM), c) **C1** (0.5 mM) and **CHDA** (1.0 mM), and d) **CHDA** (0.5 mM).

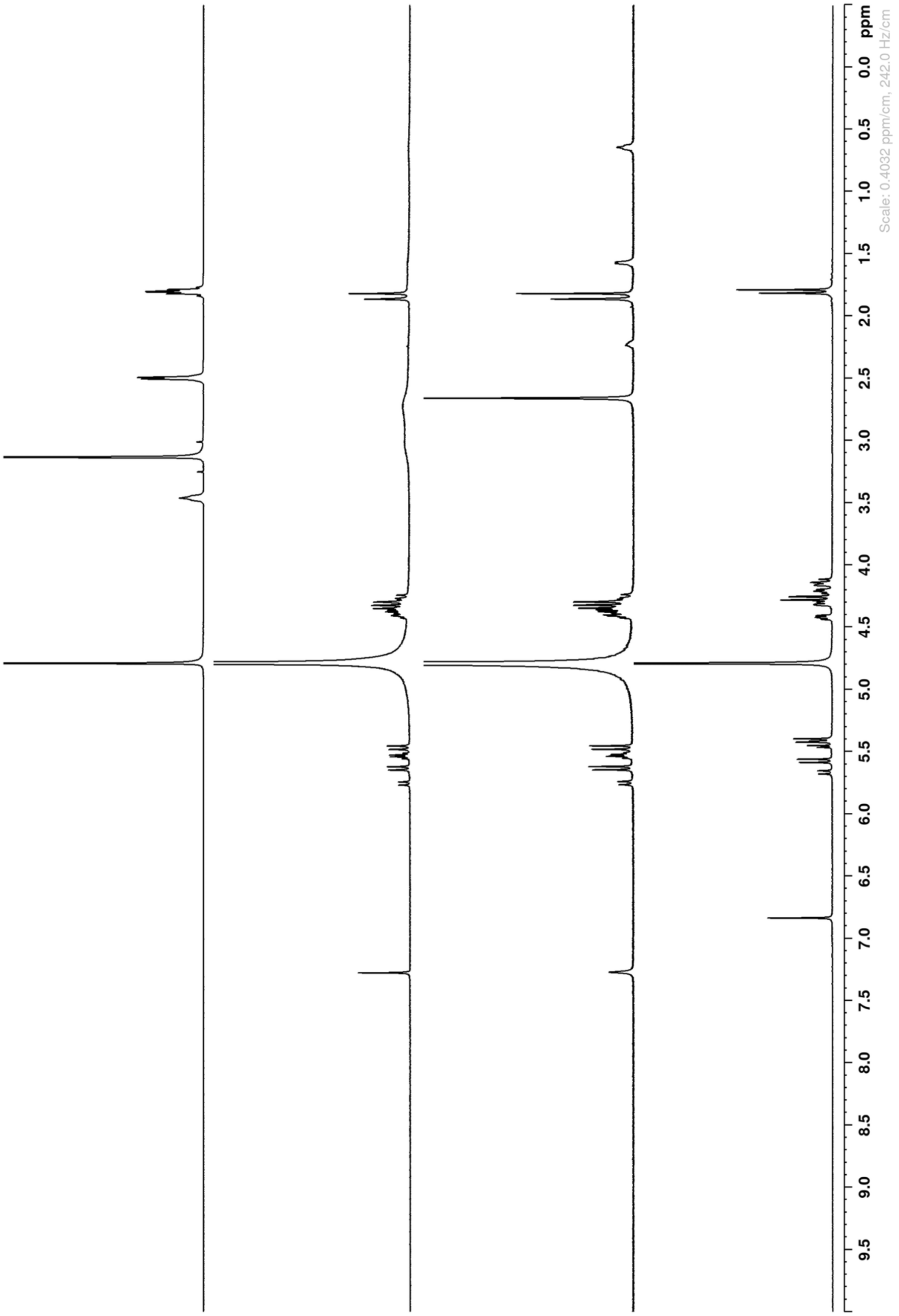

Figure S11. ^{1}H NMR spectra (400 MHz, D_2O, rt) recorded for: a) **C1** (0.5 mM), b) **C1** (0.5 mM) and **Me_6CHDA** (0.5 mM), c) **C1** (0.5 mM) and **Me_6CHDA** (1.0 mM), and d) **Me_6CHDA** (0.5 mM), (400 MHz, D_2O).

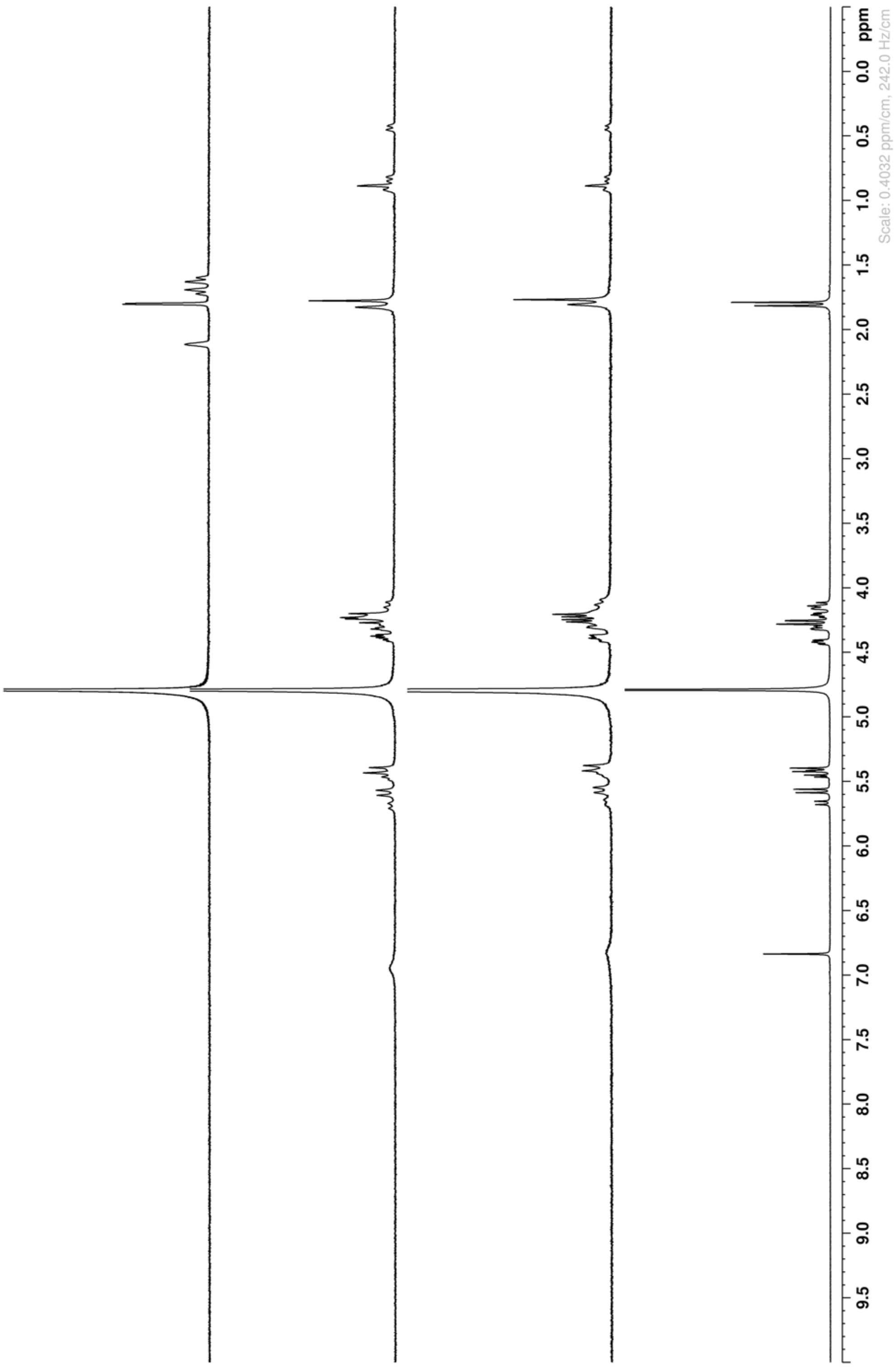

Figure S12. ^{1}H NMR spectra (600 MHz, D_2O, rt) recorded for: a) **C1** (0.5 mM), b) **C1** (0.5 mM) and **AdA** (0.5 mM), c) **C1** (0.5 mM) and **AdA** (1.0 mM), and d) **AdA** (0.5 mM).

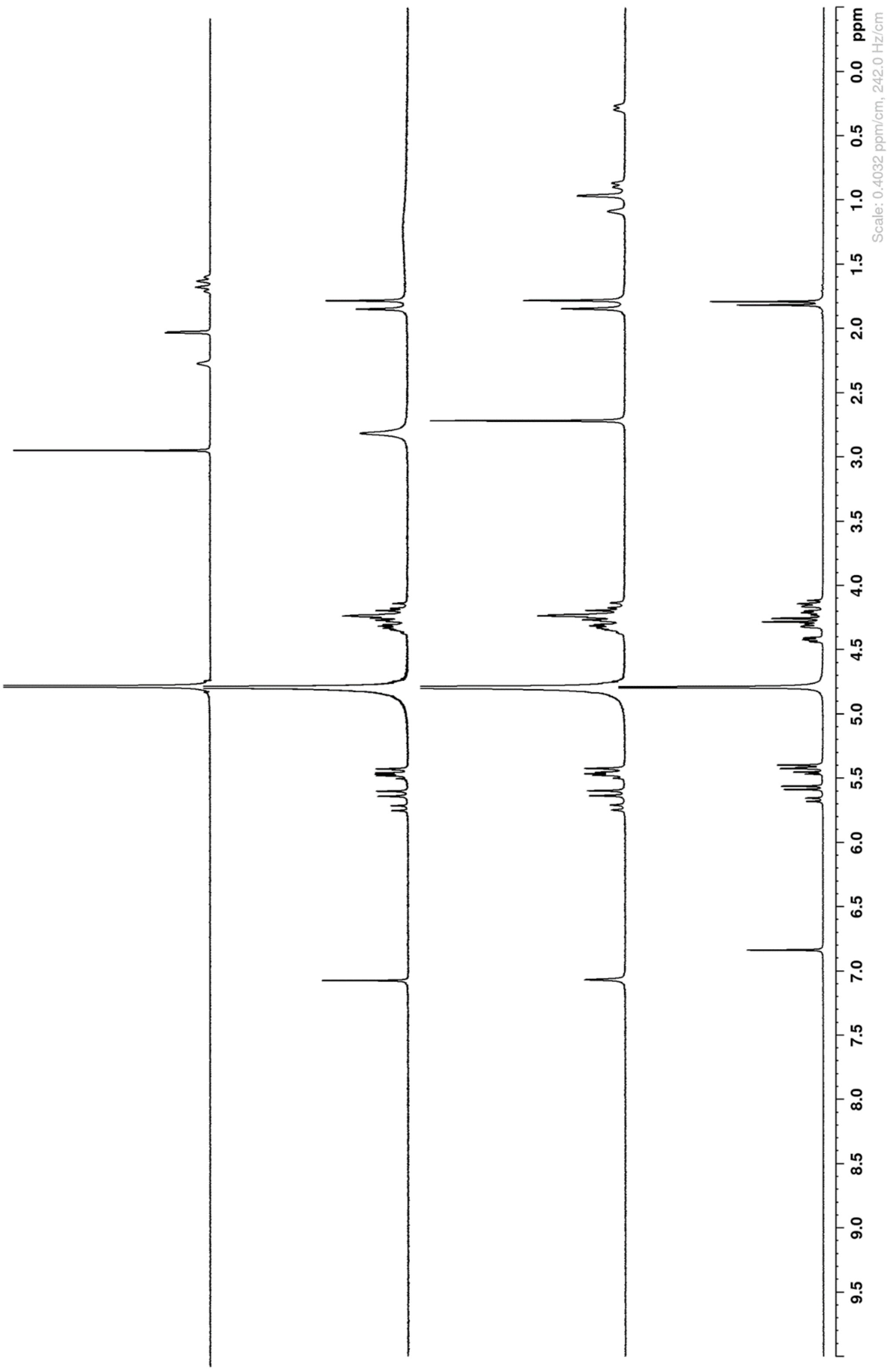

Figure S13. ^{1}H NMR spectra (400 MHz, D_2O, rt) recorded for: a) **C1** (0.5 mM), b) **C1** (0.5 mM) and **Me_3AdA** (0.5 mM), c) **C1** (0.5 mM) and **Me_3AdA** (1.0 mM), and d) **Me_3AdA** (0.5 mM).

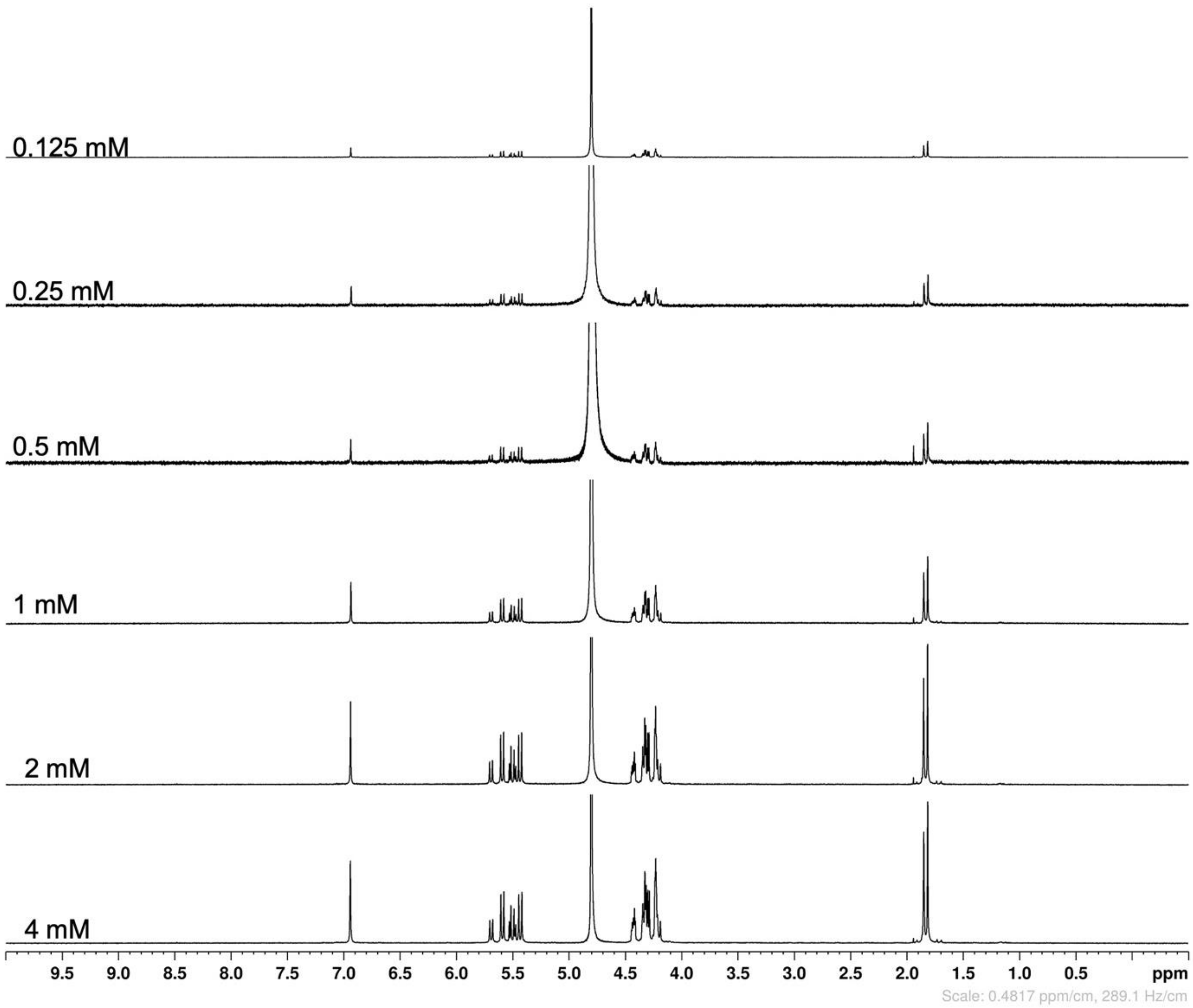

Figure S14. ^{1}H NMR spectra recorded at different concentrations of **C1** (400 MHz, PBS, rt).

Isothermal titration calorimetry experiments for C1

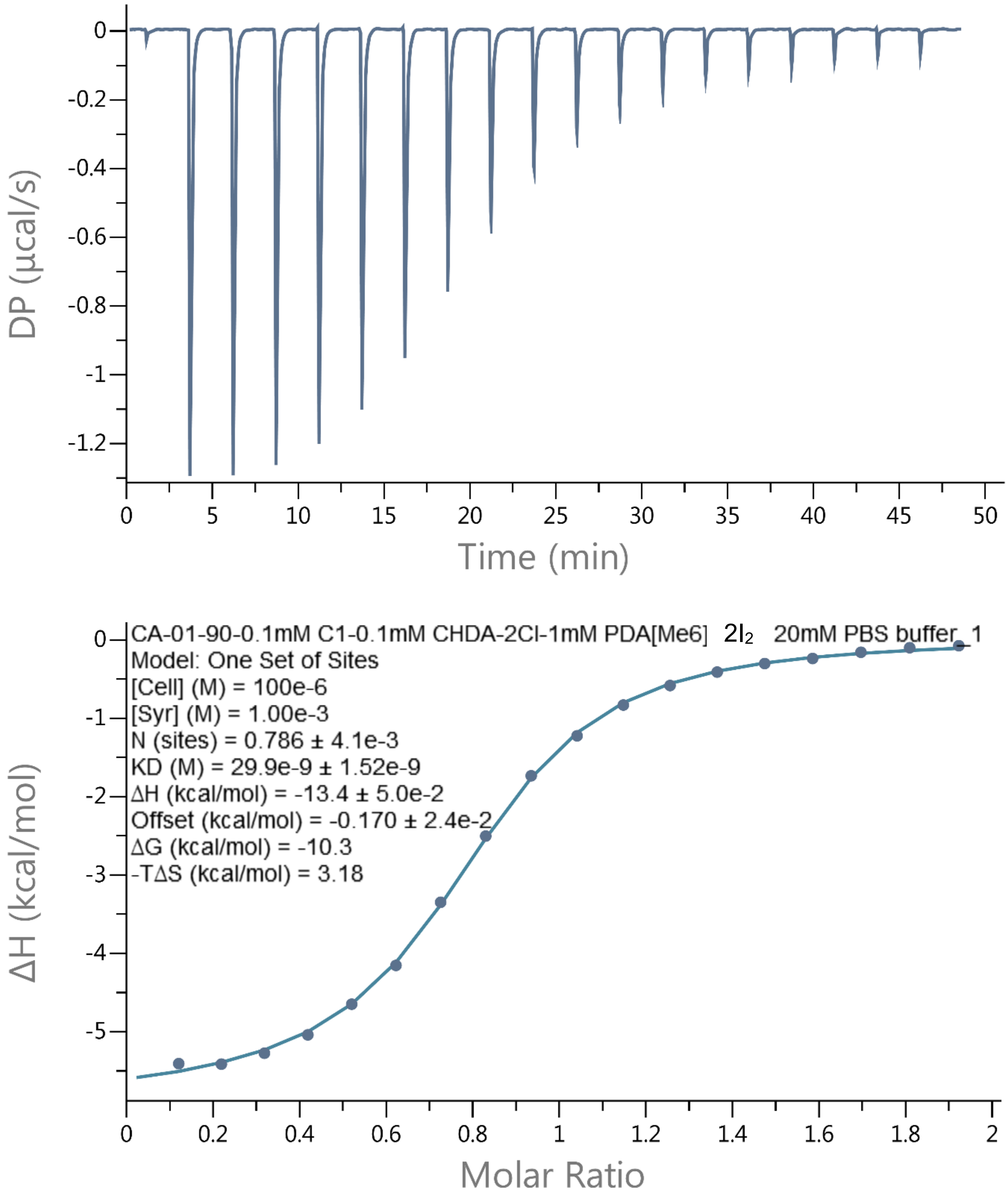

Figure S15. Isothermal titration calorimetry (ITC) curve obtained through competition binding titration studies. A solution of **C1** (0.1 mM) and **CHDA** (0.1 mM) in the cell was titrated with **Me₆PDA** (1.00 mM) from the syringe at 298.0 K in PBS buffer at pH 7.4. The data was fitted to a competitive binding model to extract $K_a = (3.40 \pm 0.09) \times 10^7$ M^{-1} and $\Delta H = (-10.13 \pm 0.02)$ kcal mol^{-1}.

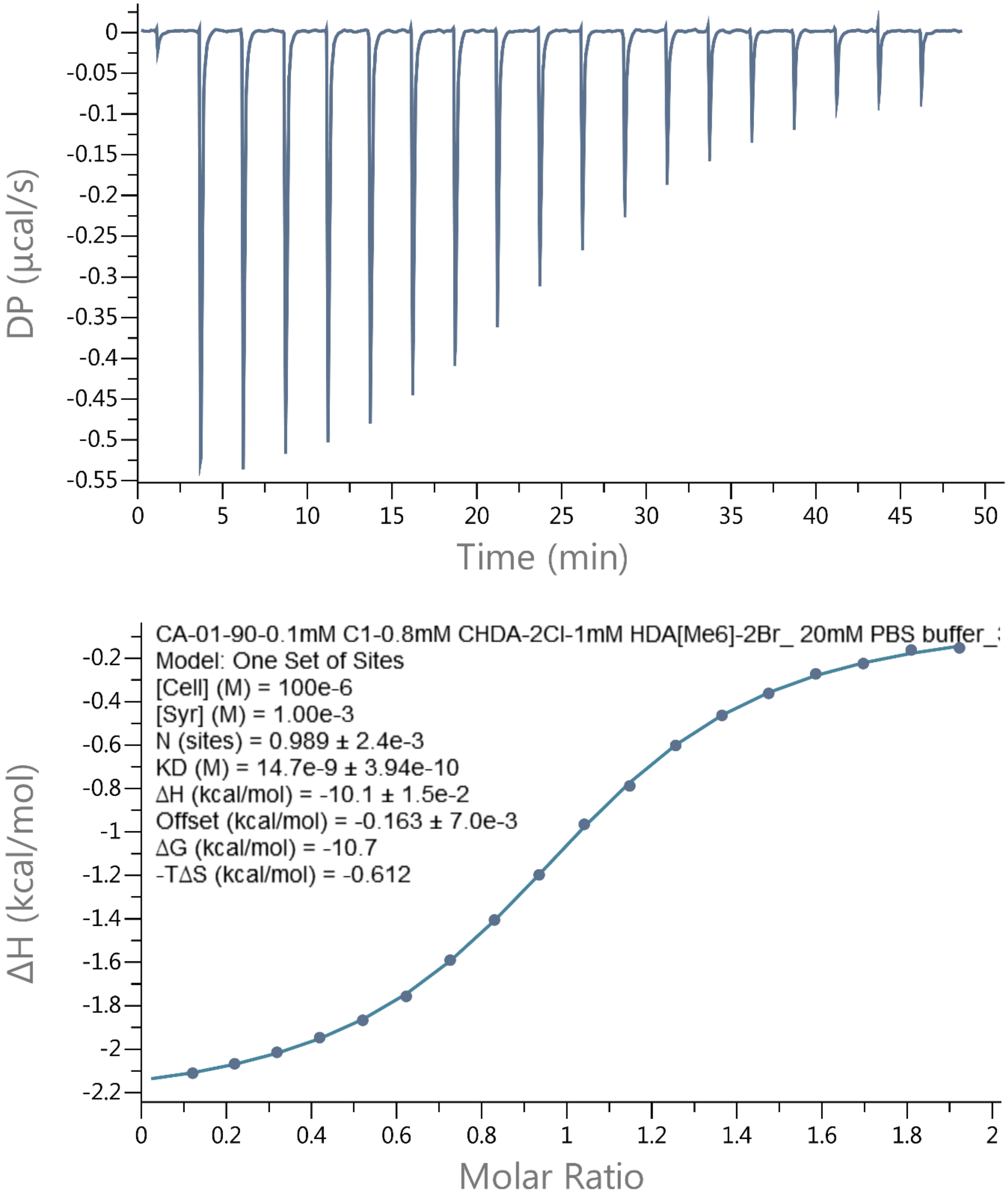

Figure S16. Isothermal titration calorimetry (ITC) curve obtained through competition binding titration studies. A solution of **C1** (0.1 mM) and **CHDA** (0.8 mM) in the cell was titrated with **Me_6HDA** (1.00 mM) from the syringe at 298.0 K in PBS buffer at pH 7.4. The data was fitted to a competitive binding model to extract $K_a = (6.54 \pm 0.59) \times 10^7$ M^{-1} and $\Delta H = (-10.13 \pm 0.02)$ kcal mol^{-1}.

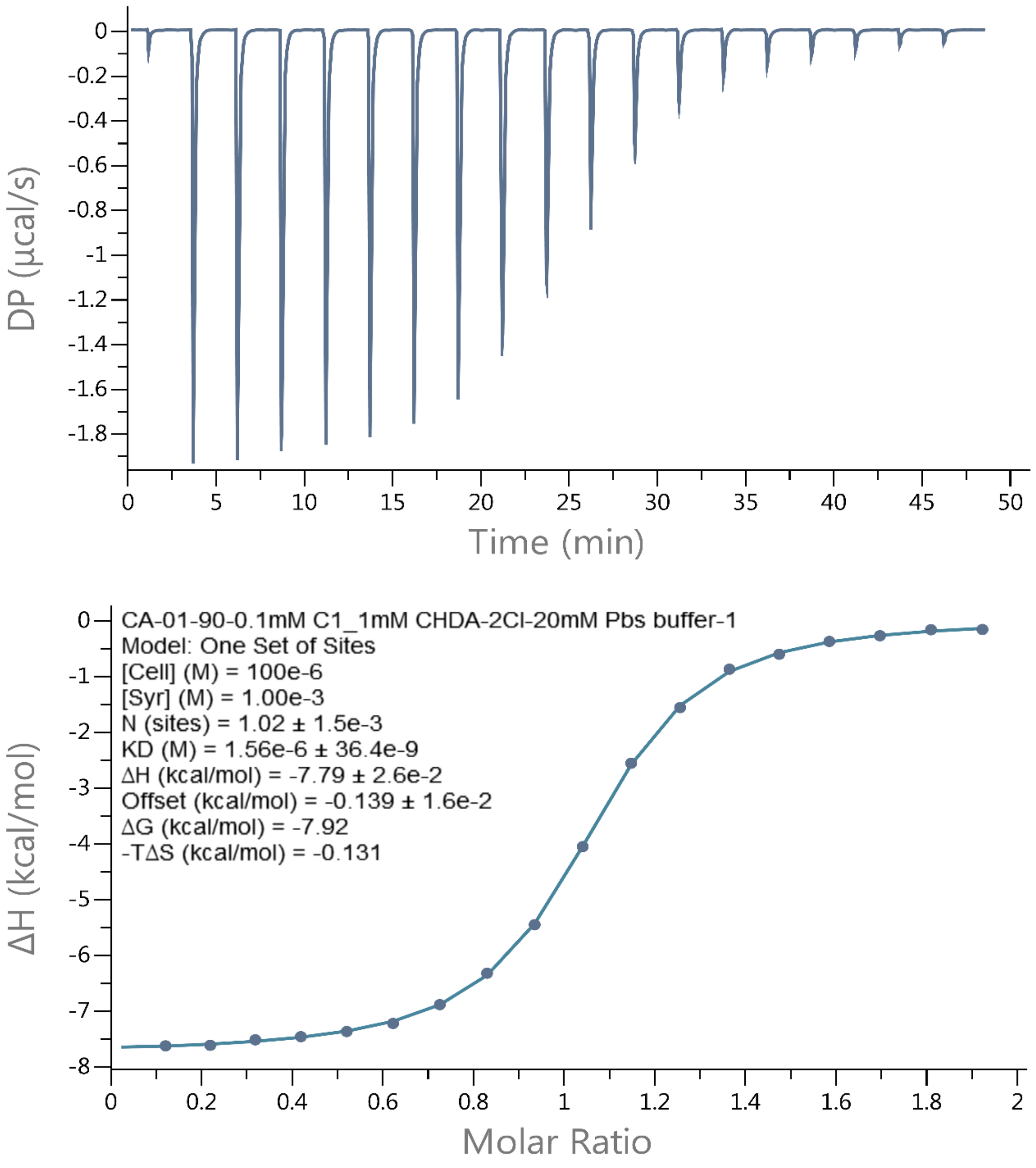

Figure S17. Isothermal titration calorimetry (ITC) curve obtained through direct binding titration studies. A solution of **C1** (100 µM) in the cell was titrated with **CHDA** (1.00 mM) in the syringe at 298.0 K in PBS at pH 7.4. The data was fitted to the single set of sites binding model to extract $K_a = (6.48 \pm 0.10) \times 10^5\ M^{-1}$ and $\Delta H = (-7.82 \pm 0.02)$ kcal mol^{-1}.

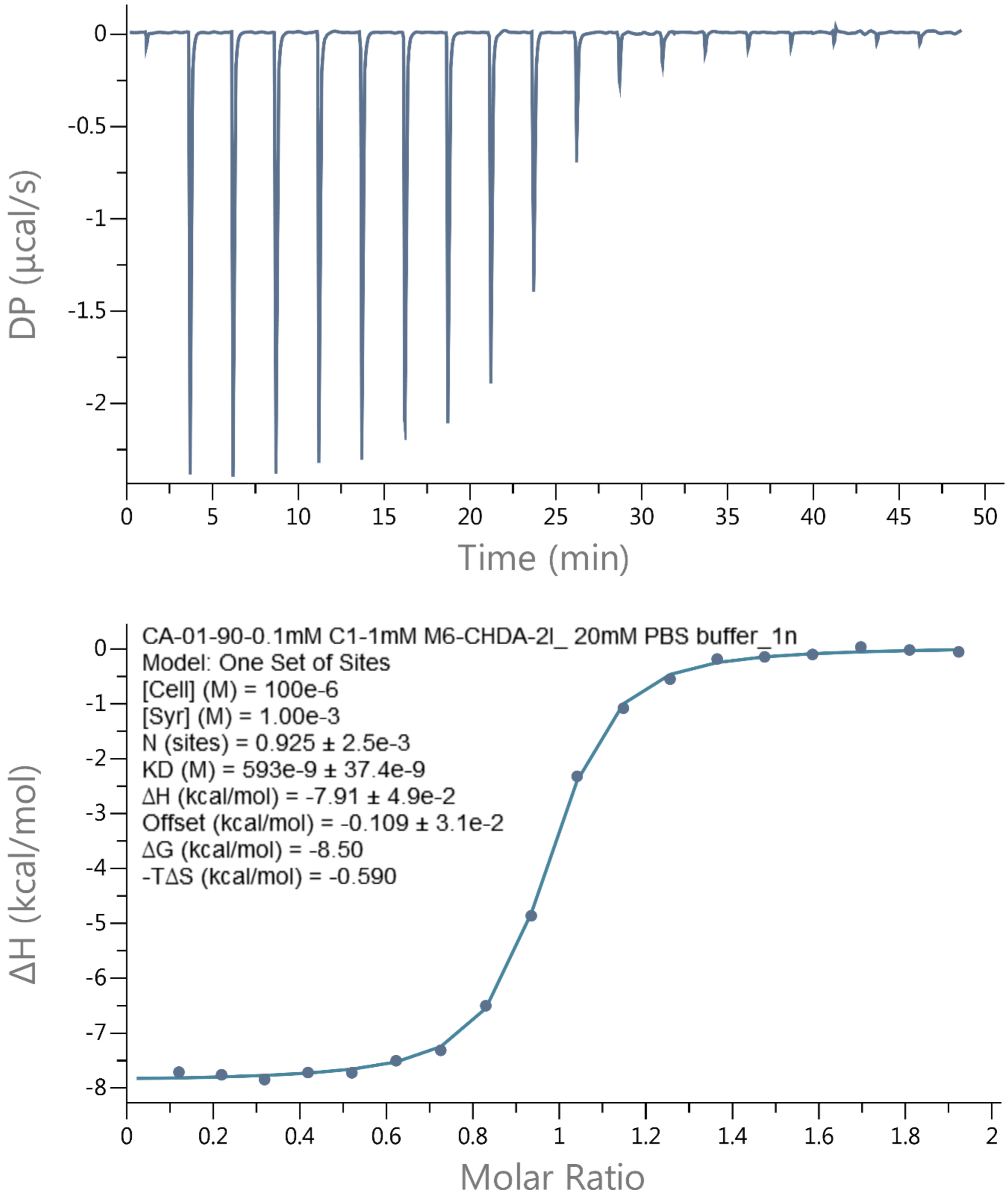

Figure S18. Isothermal titration calorimetry (ITC) curve obtained through direct binding titration studies. A solution of **C1** (100 μM) in the cell was titrated with **Me$_6$CHDA** (1.00 mM) in the syringe at 298.0 K in PBS at pH 7.4. The data was fitted to the single set of sites binding model to extract $K_a = (1.75 \pm 0.06) \times 10^6$ M^{-1} and $\Delta H = (-7.83 \pm 0.03)$ kcal mol^{-1}.

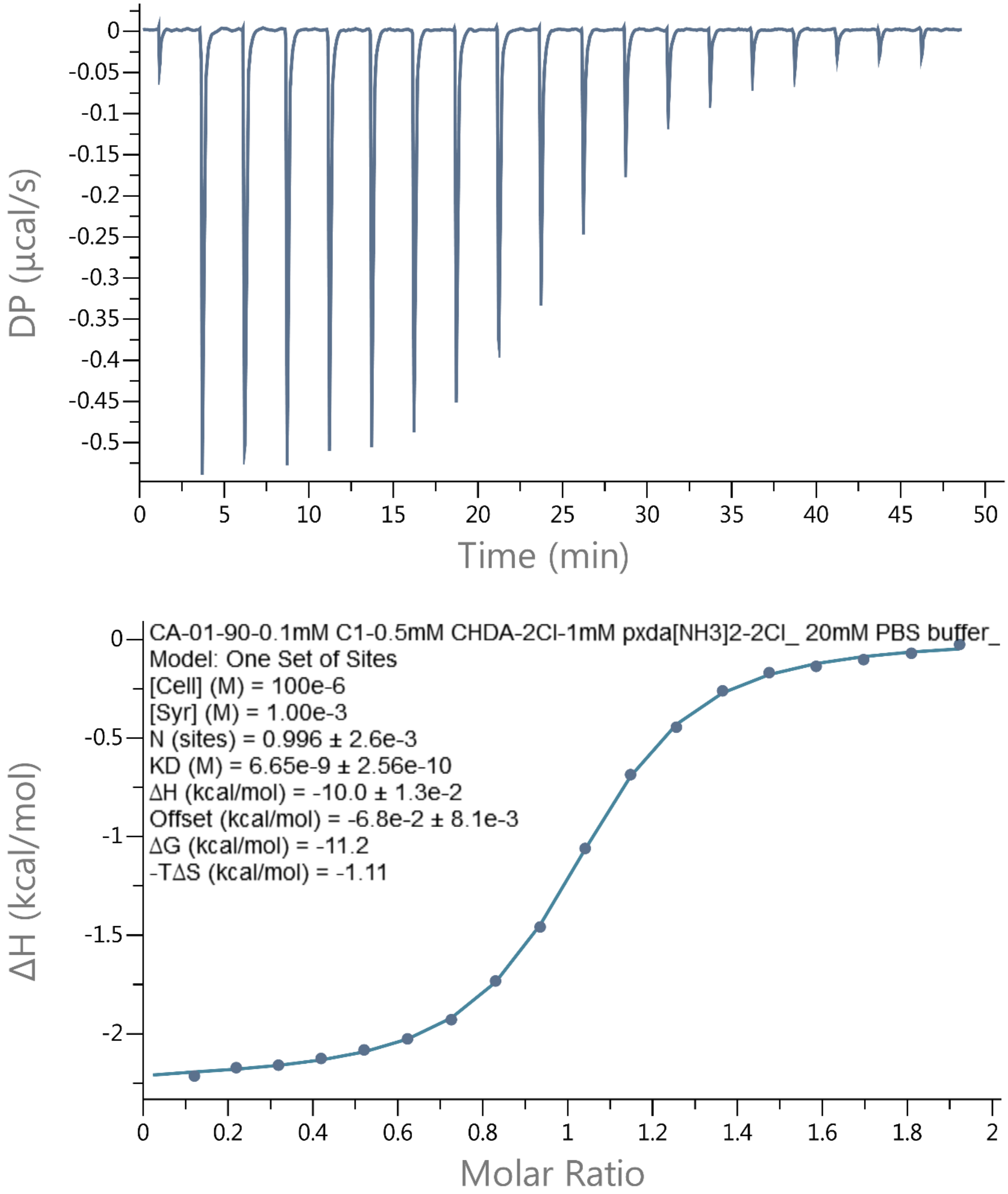

Figure S19. Isothermal titration calorimetry (ITC) curve obtained through competition binding titration studies. A solution of **C1** (0.1 mM) and **CHDA** (0.5 mM) in the cell was titrated with **PXDA** (1.00 mM) from the syringe at 298.0 K in PBS buffer at pH 7.4. The data was fitted to a competitive binding model to extract $K_a = (1.44 \pm 0.03) \times 10^8$ M^{-1} and $\Delta H = (-10.07 \pm 0.01)$ kcal mol^{-1}.

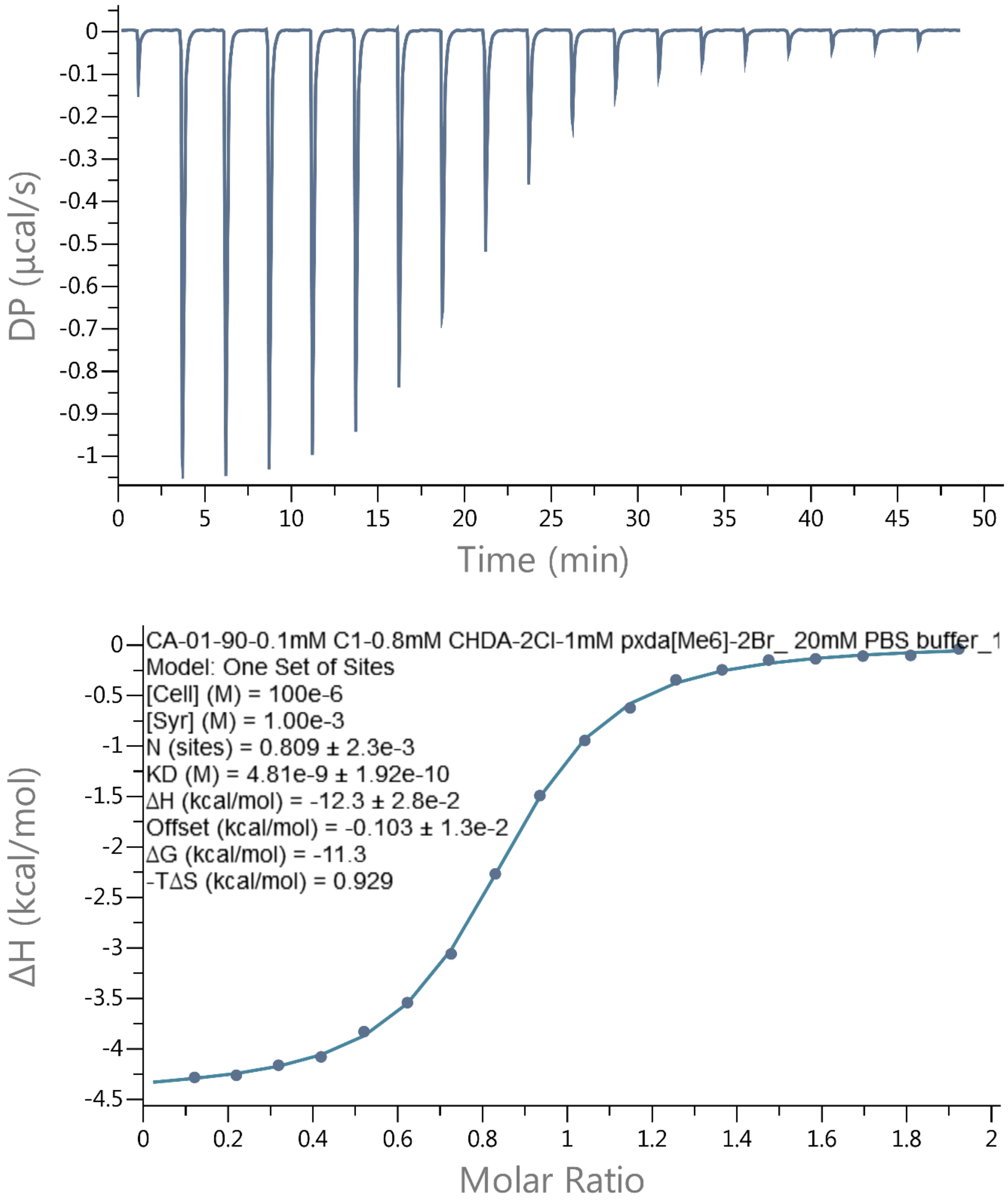

Figure S20. Isothermal titration calorimetry (ITC) curve obtained through competition binding titration studies. A solution of **C1** (0.1 mM) and **CHDA** (0.8 mM) in the cell was titrated with **Me$_6$PXDA** (1.00 mM) in the syringe at 298.0 K in 20 mM phosphate buffered water at pH 7.4. The data was fitted to a competitive binding model to extract $K_a = (2.47 \pm 0.06) \times 10^8$ M^{-1} and $\Delta H = (-12.43 \pm 0.02)$ kcal mol^{-1}.

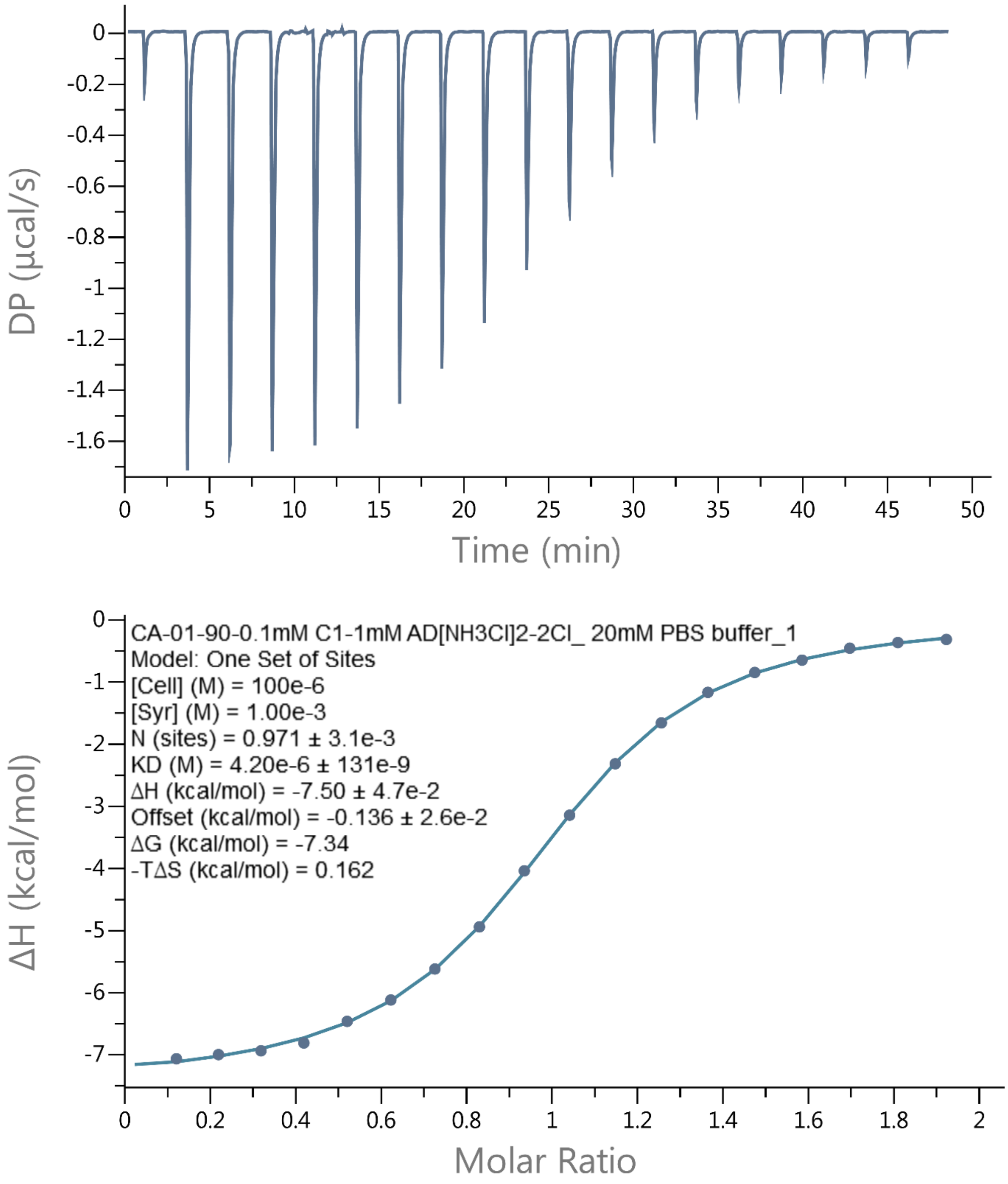

Figure S21. Isothermal titration calorimetry (ITC) curve obtained through direct binding titration studies. A solution of **C1** (100 µM) in the cell was titrated with **AdA** (1.00 mM) in the syringe at 298.0 K in PBS buffer at pH 7.4. The data was fitted to the single set of sites binding model to extract $K_a = (2.41 \pm 0.04) \times 10^5$ M^{-1} and $\Delta H = (-7.54 \pm 0.03)$ kcal mol^{-1}.

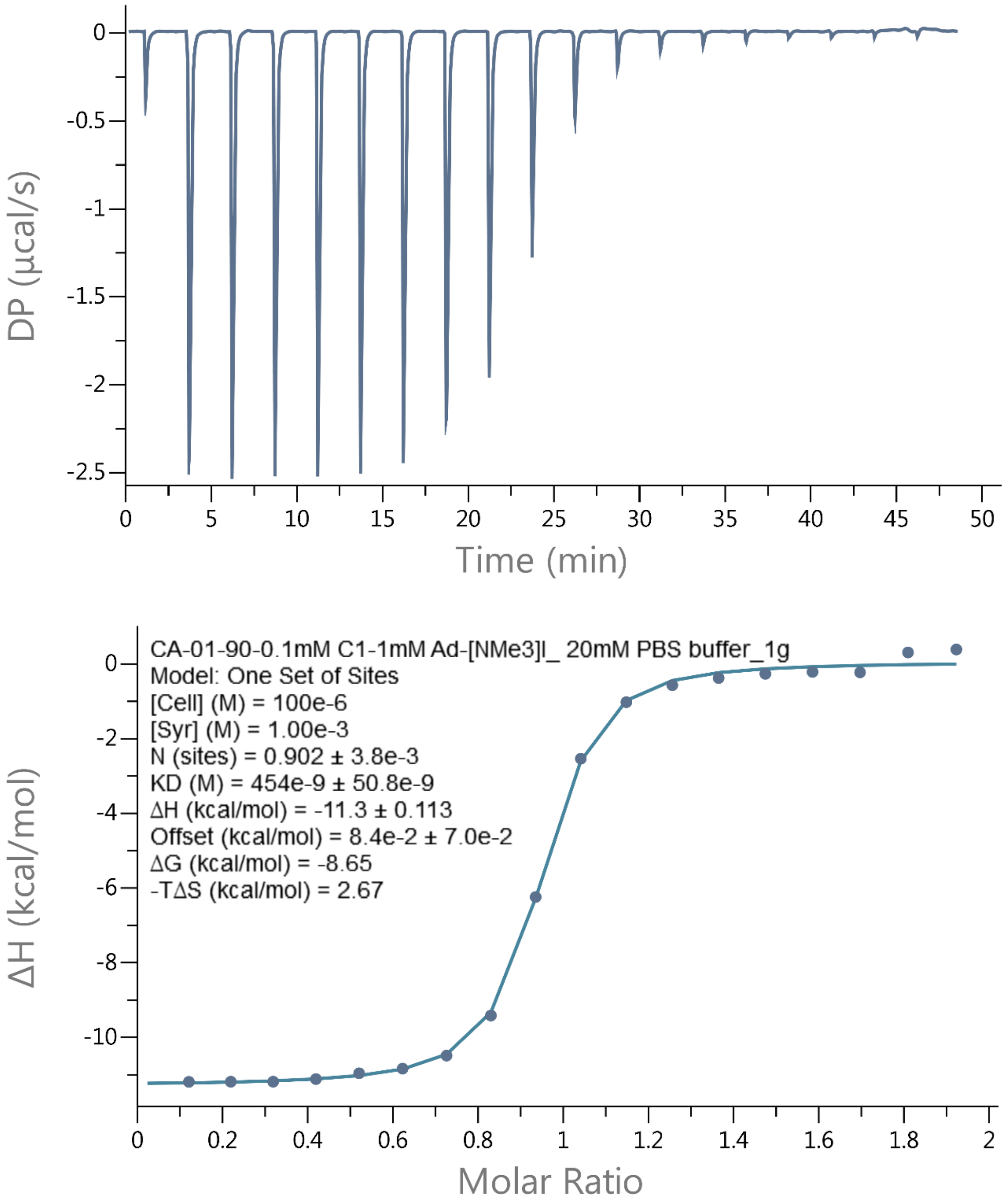

Figure S22. Isothermal titration calorimetry (ITC) curve obtained through direct binding titration studies. A solution of **C1** (0.1 mM) in the cell was titrated with **Me$_3$AdA** (1.00 mM) in the syringe at 298.0 K in PBS buffer at pH 7.4. The data was fitted to the single set of sites binding model to extract $K_a = (2.31 \pm 0.10) \times 10^6$ M^{-1} and $\Delta H = (-9.80 \pm 0.04)$ kcal mol^{-1}.

Isothermal titration calorimetry study of M1

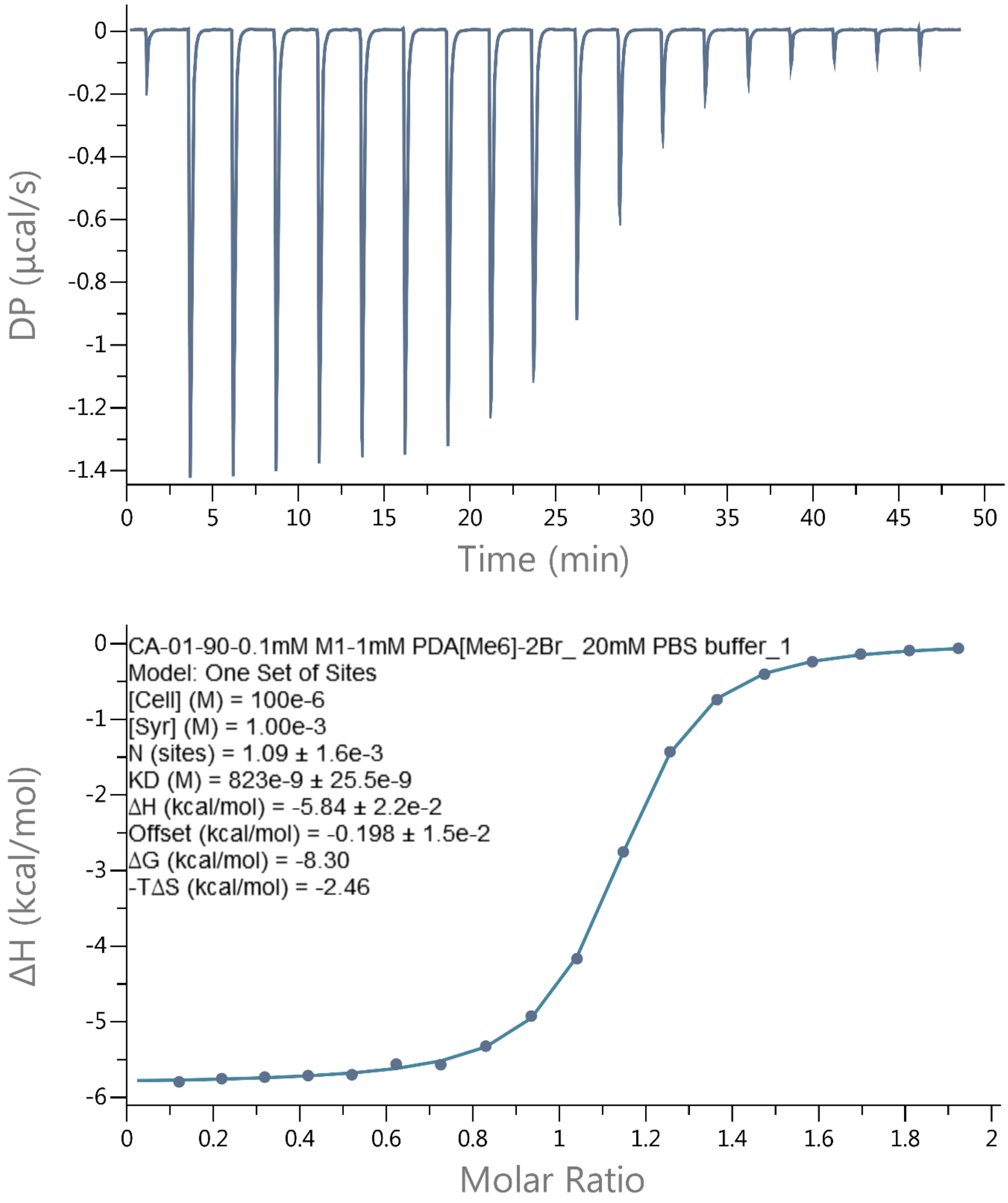

Figure S23. Isothermal titration calorimetry (ITC) curve obtained through direct binding titration studies. A solution of **M1** (0.1 mM) in the cell was titrated with **Me₆PDA** (1.00 mM) from the syringe at 298.0 K in PBS buffer at pH 7.4. The data was fitted to the single set of sites binding model to extract $K_a = (1.31 \pm 0.05) \times 10^6\ M^{-1}$ and $\Delta H = (-5.98 \pm 0.03)$ kcal mol^{-1}.

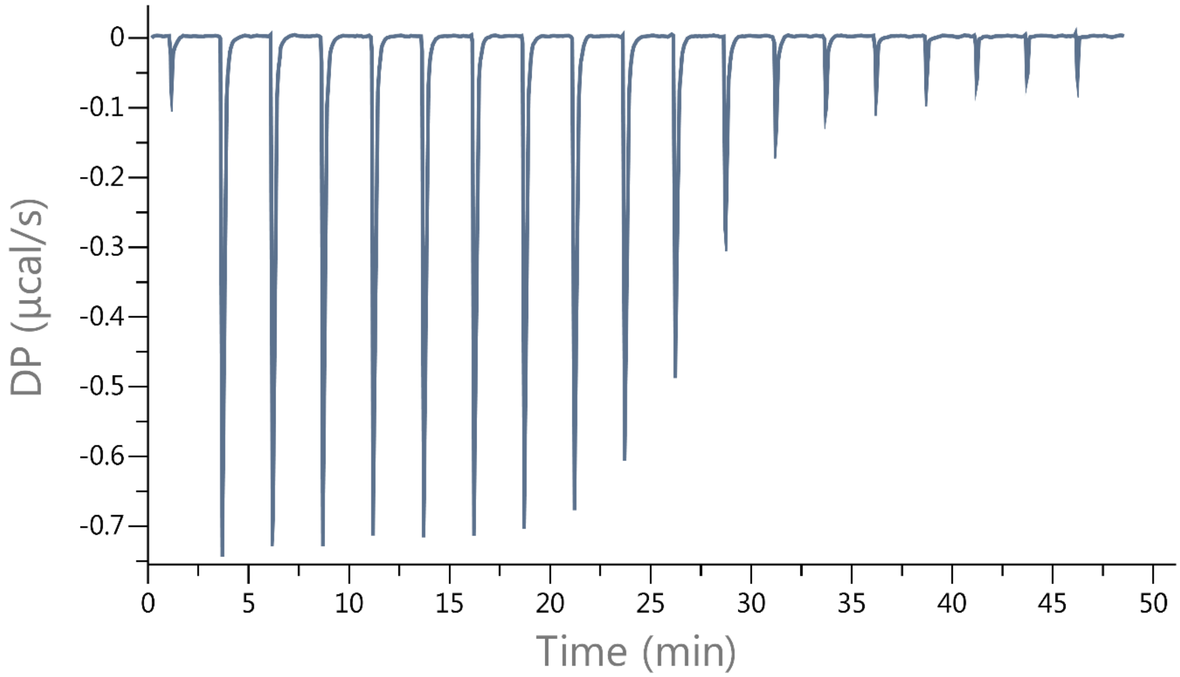

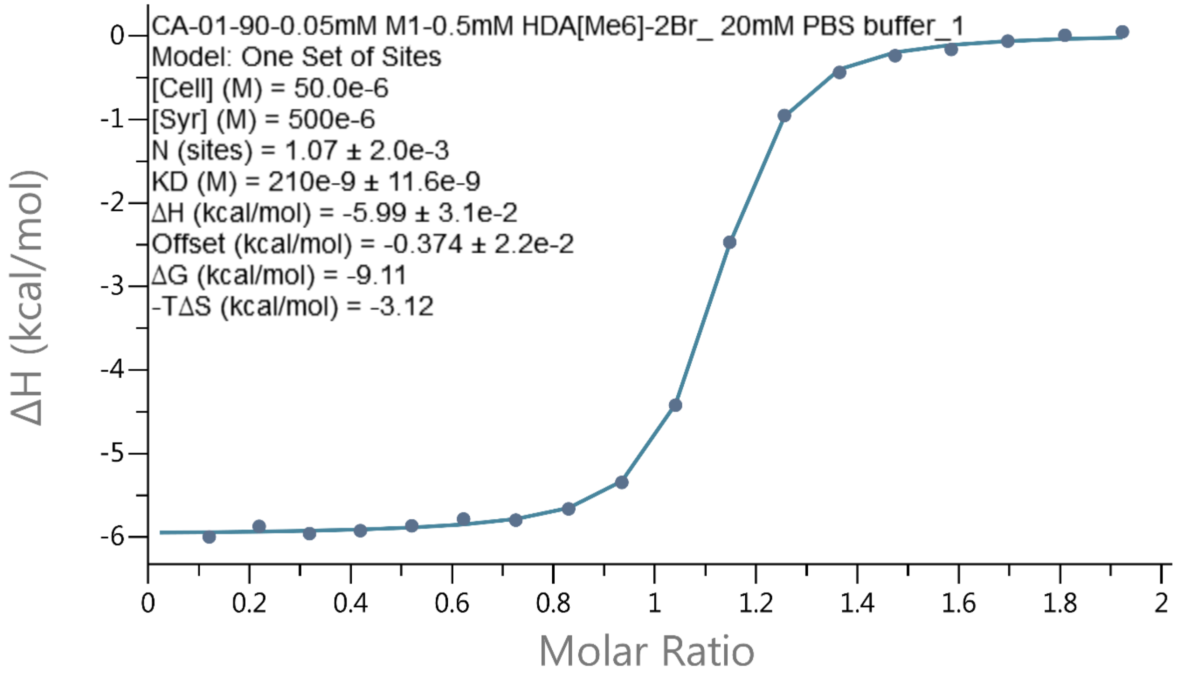

Figure S24. Isothermal titration calorimetry (ITC) curve obtained through direct binding titration studies. A solution of **M1** (0.05 mM) in the cell was titrated with **Me$_6$HDA** (0.5 mM) from the syringe at 298.0 K in PBS buffer at pH 7.4. The data was fitted to the single set of sites binding model to extract $K_a = (4.37 \pm 0.22) \times 10^6$ M^{-1} and $\Delta H = (-6.14 \pm 0.03)$ kcal mol^{-1}.

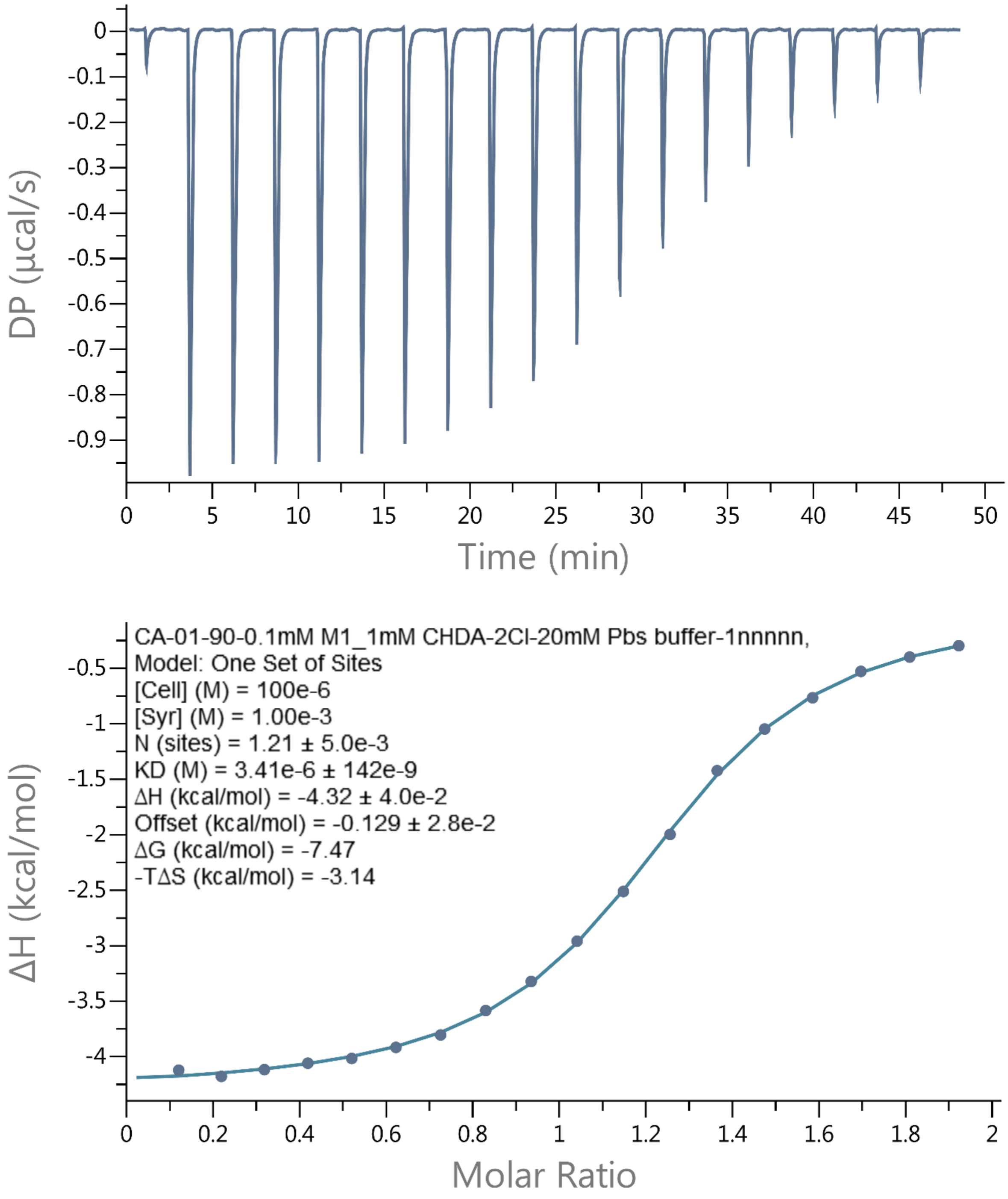

Figure S25. Isothermal titration calorimetry (ITC) curve obtained through direct binding titration studies. A solution of **M1** (100 µM) in the cell was titrated with **CHDA** (1.00 mM) from the syringe at 298.0 K in PBS buffer at pH 7.4. The data was fitted to the single set of sites binding model to extract $K_a = (2.79 \pm 0.07) \times 10^5$ M^{-1} and $\Delta H = (-4.38 \pm 0.02)$ kcal mol^{-1}.

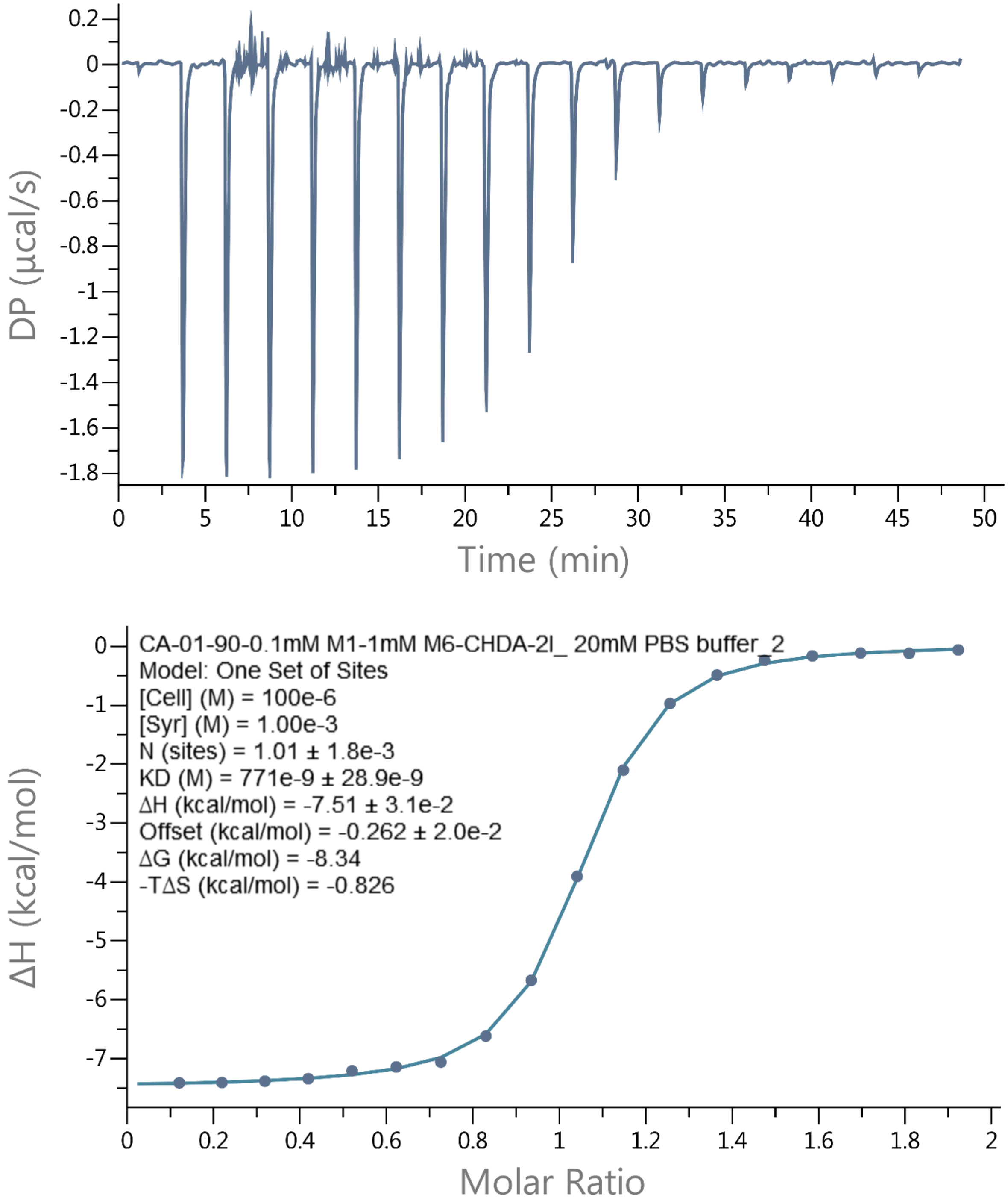

Figure S26. Isothermal titration calorimetry (ITC) curve obtained through direct binding titration studies. A solution of **M1** (100 µM) in the cell was titrated with **Me$_6$CHDA** (1.00 mM) from the syringe at 298.0 K in PBS at pH 7.4. The data was fitted to the single set of sites binding model to extract $K_a = (1.20 \pm 0.03) \times 10^6$ M^{-1} and $\Delta H = (-7.44 \pm 0.03)$ kcal mol^{-1}.

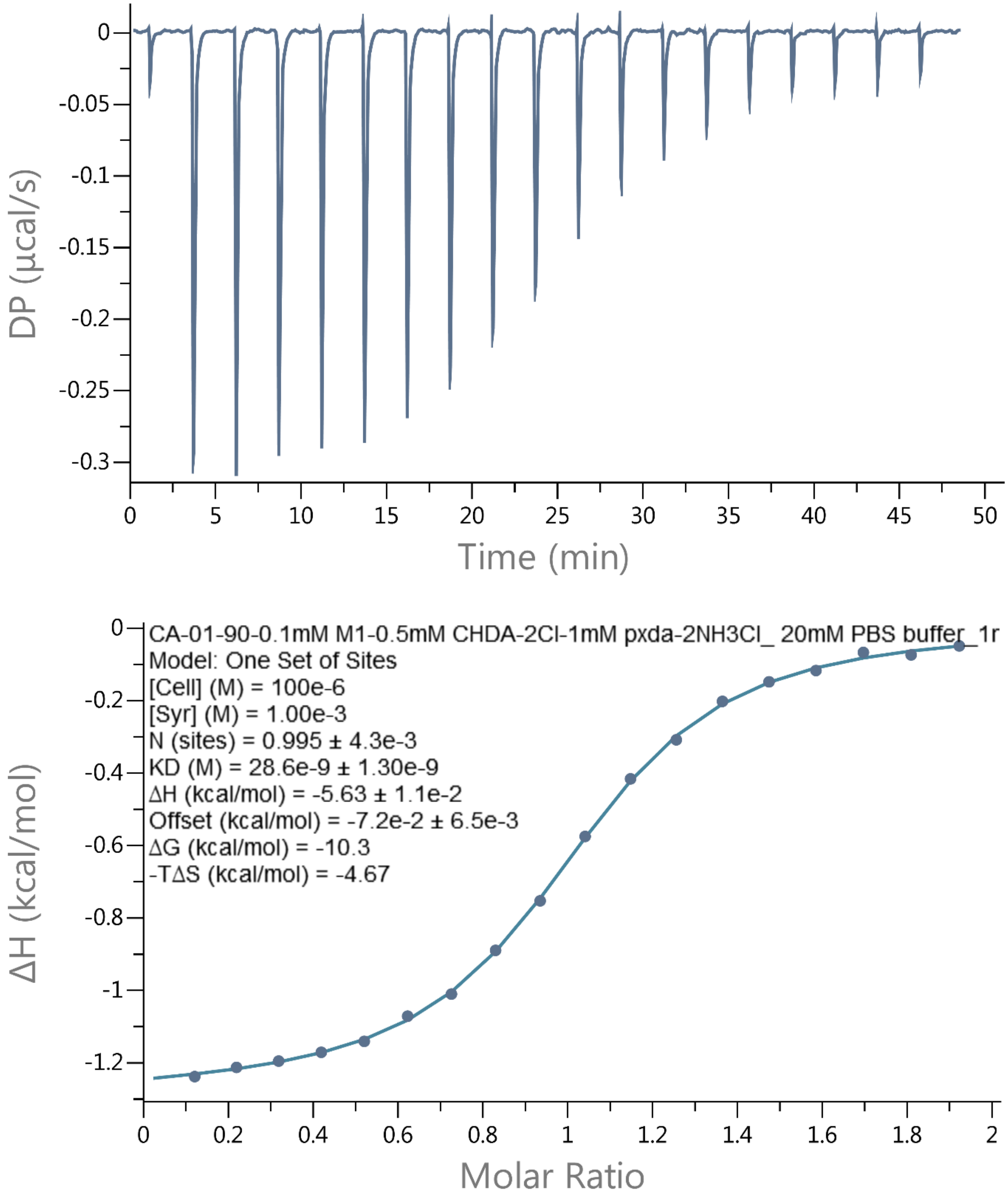

Figure S27. Isothermal titration calorimetry (ITC) curve obtained through competition binding titration studies. A solution of **M1** (0.1 mM) and **CHDA** (0.5 mM) in the cell was titrated with **PXDA** (1.00 mM) from the syringe at 298.0 K in PBS buffer at pH 7.4. The data was fitted to a competitive binding model to extract $K_a = (3.42 \pm 0.05) \times 10^7$ M^{-1} and $\Delta H = (-5.67 \pm 0.01)$ kcal mol^{-1}.

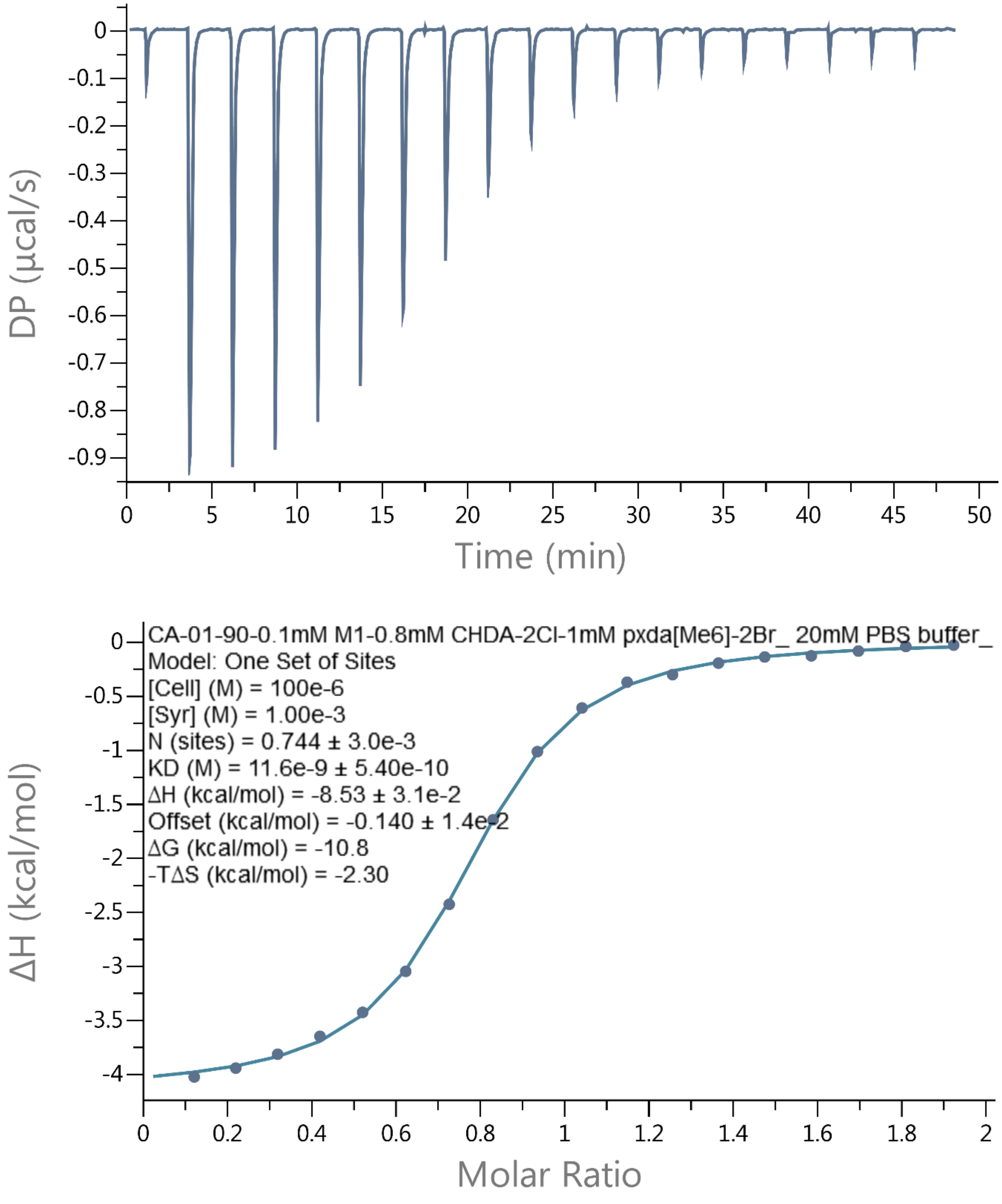

Figure S28. Isothermal titration calorimetry (ITC) curve obtained through competition binding titration studies. A solution of **M1** (0.1 mM) and **CHDA** (0.8 mM) in the cell was titrated with **Me$_6$PXDA** (1.00 mM) from the syringe at 298.0 K in PBS buffer at pH 7.4. The data was fitted to a competitive binding model to extract $K_a = (7.52 \pm 0.18) \times 10^7$ M^{-1} and $\Delta H = (-8.64 \pm 0.02)$ kcal mol^{-1}.

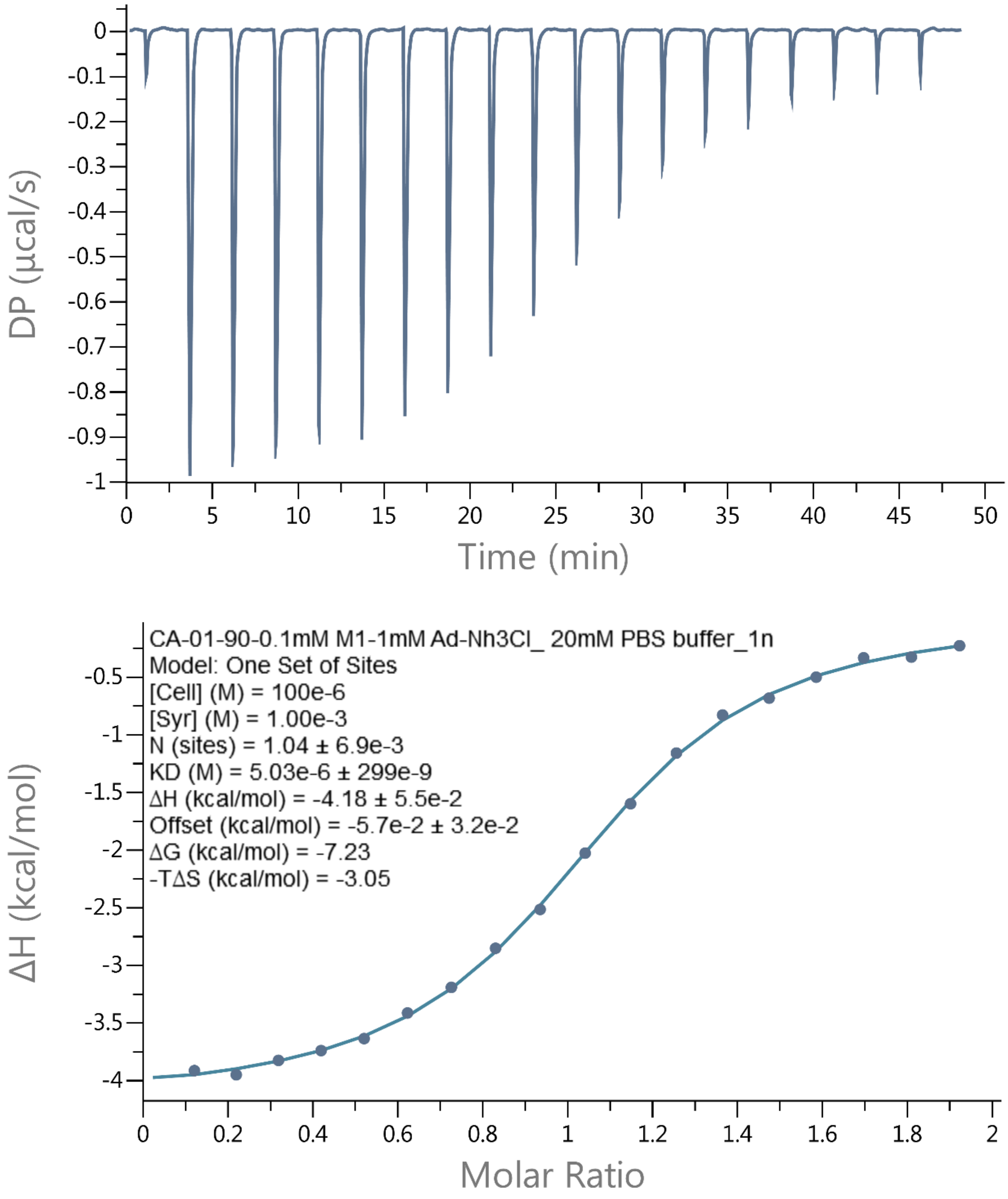

Figure S29. Isothermal titration calorimetry (ITC) curve obtained through direct binding titration studies. A solution of **M1** (100 µM) in the cell was titrated with **AdA** (1.00 mM) from the syringe at 298.0 K in PBS buffer water at pH 7.4. The data was fitted to the single set of sites binding model to extract $K_a = (1.99 \pm 0.06) \times 10^5\ M^{-1}$ and $\Delta H = (-4.11 \pm 0.03)$ kcal mol^{-1}.

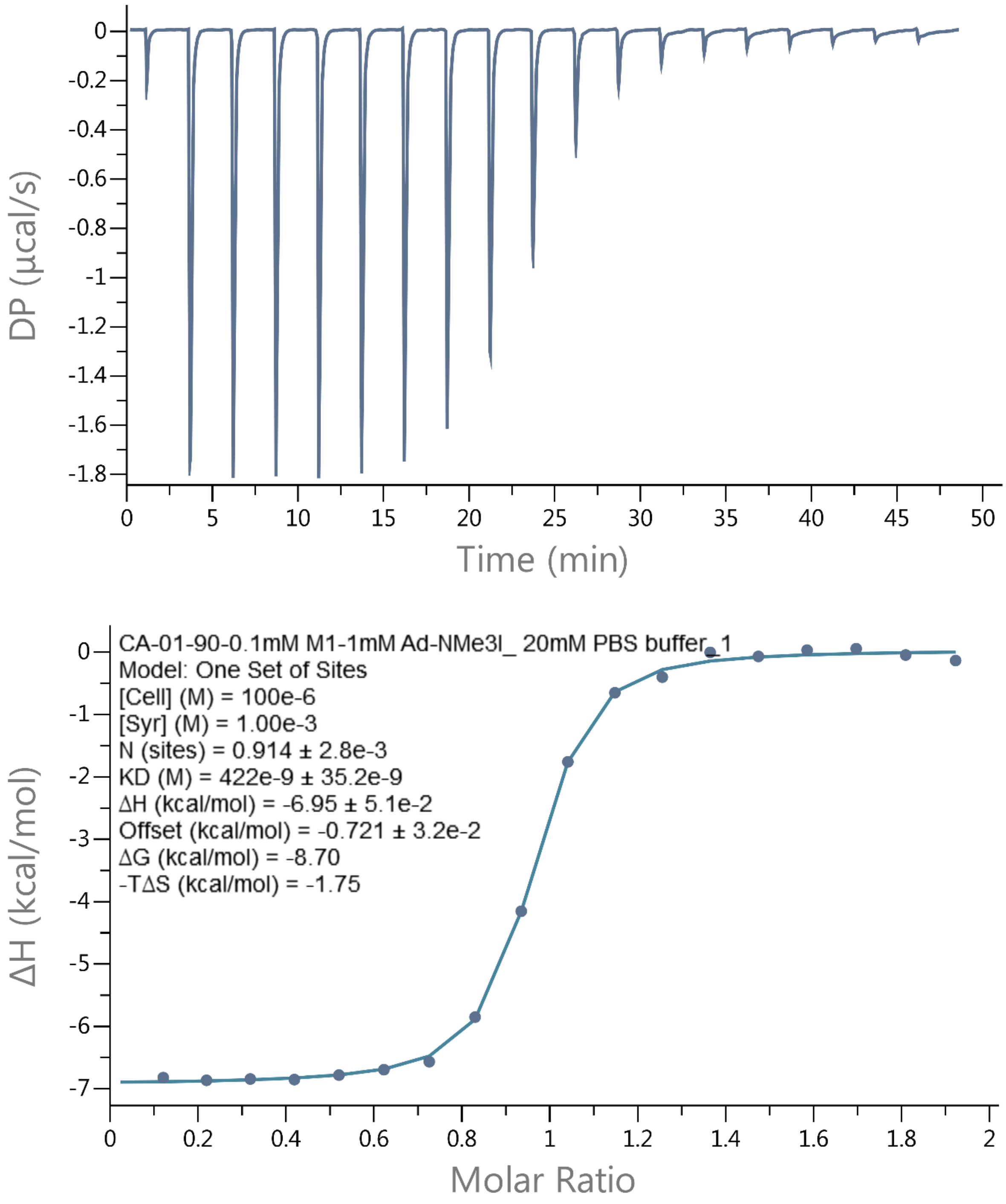

Figure S30. Isothermal titration calorimetry (ITC) curve obtained through direct binding titration studies. A solution of **M1** (0.1 mM) in the cell was titrated with **Me$_3$AdA** (1.00 mM) from the syringe at 298.0 K in PBS buffer at pH 7.4. The data was fitted to the single set of sites binding model to extract $K_a = (2.09 \pm 0.07) \times 10^6$ M^{-1} and $\Delta H = (-7.42 \pm 0.02)$ kcal mol^{-1}.
